# The effect of medium supplementation and serial passaging on the transcriptome of human adipose-derived stromal cells expanded in vitro

**DOI:** 10.1186/s13287-019-1370-2

**Published:** 2019-08-14

**Authors:** Carla Dessels, Melvin A. Ambele, Michael S. Pepper

**Affiliations:** 1 Department of Immunology, Institute for Cellular and Molecular Medicine, SAMRC Extramural Unit for Stem Cell Research and Therapy, Faculty of Health Sciences, University of Pretoria, PO Box 2034, Pretoria, 0001 South Africa; 2 Department of Oral Pathology and Oral Biology, School of Dentistry, Faculty of Health Sciences, University of Pretoria, PO Box 1266, Pretoria, 0001 South Africa

**Keywords:** Adipose-derived stromal cells, Pooled human platelet lysate, Fetal bovine serum, Transcriptome

## Abstract

**Background:**

For adipose-derived stromal cells (ASCs) to be safe for use in the clinical setting, they need to be prepared using good manufacturing practices (GMPs). Fetal bovine serum (FBS), used to expand ASCs in vitro in some human clinical trials, runs the risk of xenoimmunization and zoonotic disease transmission. To ensure that GMP standards are maintained, pooled human platelet lysate (pHPL) has been used as an alternative to FBS. ASCs proliferate more rapidly in pHPL than in FBS, with no significant change in immunophenotype and differentiation capacity. However, not much is known about how pHPL affects the transcriptome of these cells.

**Methods:**

This study investigated the effect of pHPL and FBS on the ASC transcriptome during in vitro serial expansion from passage 0 to passage 5 (P0 to P5). RNA was isolated from ASCs at each passage and hybridized to Affymetrix HuGene 2.0 ST arrays for gene expression analysis.

**Results:**

We observed that the transcriptome of ASCs expanded in pHPL (pHPL-ASCs) and FBS (FBS-ASCs) had the greatest change in gene expression at P2. Gene ontology revealed that genes upregulated in pHPL-ASCs were enriched for cell cycle, migration, motility, and cell-cell interaction processes, while those in FBS-ASCs were enriched for immune response processes. ASC transcriptomes were most homogenous from P2 to P5 in FBS and from P3 to P5 in pHPL. FBS- and pHPL-gene-specific signatures were observed, which could be used as markers to identify cells previously grown in either FBS or pHPL for downstream clinical/research applications. The number of genes constituting the FBS-specific effect was 3 times greater than for pHPL, suggesting that pHPL may be a milder supplement for cell expansion. A set of genes were expressed in ASCs at all passages and in both media. This suggests that a unique ASC in vitro transcriptomic profile exists that is independent of the passage number or medium used.

**Conclusions:**

GO classification revealed that pHPL-ASCs are more involved in cell cycle processes and cellular proliferation when compared to FBS-ASCs, which are involved in more specialized or differentiation processes like cardiovascular and vascular development. This makes pHPL a potential superior supplement for expanding ASCs as they retain their proliferative capacity, remain untransformed and pHPL does not affect the genes involved in differentiation in specific developmental processes.

**Electronic supplementary material:**

The online version of this article (10.1186/s13287-019-1370-2) contains supplementary material, which is available to authorized users.

## Background

Adipose-derived stromal cells (ASCs) could constitute a novel therapeutic option for the treatment of several diseases and are increasingly being assessed in clinical trials for this purpose [1–3]. Most clinical trials make use of ASCs that have been expanded ex vivo via several rounds of passaging in order to obtain adequate cell numbers [4, 5]. In the laboratory, ASCs are traditionally expanded in medium supplemented with fetal bovine serum (FBS); however, it has been reported that ASCs expanded in FBS cause immune reactions when given to human patients [2, 6–8]. However, for these cells to be considered safe for patient use, they need to adhere to good manufacturing processes (GMPs), in which non-defined and animal-related products are eliminated [2, 9]. As a result, several investigators have moved away from using FBS and have instead investigated the use of human alternatives such as pooled human platelet lysate (pHPL) [10–12]. Most studies compare the criteria as set out by the Mesenchymal and Tissue Stem Cell Committee of the International Society for Cellular Therapy (ISCT) and International Federation of Adipose Therapeutics and Sciences (IFATS) when comparing FBS to pHPL [6, 10, 13–15]. These criteria include ASC adherence to plastic, immunophenotypic surface marker expression and the ability to differentiate into bone, fat, and cartilage [5, 13]. The use of pHPL as a medium supplement has advantages over FBS. It has thus been reported that when the cells are expanded in pHPL, their innate characteristics are unaltered and proliferation is increased during expansion [10, 12, 16]. However, it is well known that experimental conditions, such as medium supplementation, can have an effect on gene expression [15, 17–19]. It is therefore important to demonstrate that the cells are safe for use in patients by measuring the effect of the medium supplementation at the level of gene expression. In this study, we assessed the changes in ASC gene expression that occur during serial passaging by comparing cells expanded in FBS versus pHPL.

## Material and methods

### ASC isolation and expansion

Lipoaspirate samples were collected from five individual patients undergoing elective liposuction. Stromal vascular fraction (SVF) was isolated from lipoaspirates using previously established protocols [5, 20]. SVF containing ASCs was seeded at a density of 5 × 10^5^ cells/cm^2^ in T80 flasks (80 cm^2^; NUNC™, Roskilde Site, Kamstrupvej, Denmark) and maintained in α-MEM containing 2% (v/v) penicillin [10,000 U/mL]-streptomycin [10,000 8 μg/mL] (p/s; GIBCO, Life Technologies™, New York, USA) and either 10% (v/v) fetal bovine serum (FBS; GIBCO, Life Technologies™, New York, USA) or 10% pooled human platelet lysate (pHPL) supplemented with preservative-free heparin ([2 U/mL]; Biochrom, Merck Millipore, Berlin, Germany). pHPL was manufactured as previously described in our laboratory and subjected to quality control checks [21, 22]. At 80 to 90% confluence, ASCs were dissociated using trypLE (Life Technologies™, New York, USA) and counted. ASCs at passage zero (P0) were expanded by plating 5 × 10^3^ cells/cm^2^ into T80 flasks and were maintained in α-MEM containing 2% (v/v) p/s and either 10% (v/v) pHPL or 10% (v/v) FBS at 37 °C in 5% CO_2_. The passaging process was repeated from P0 to P5 for ASCs expanded in FBS and pHPL. ASCs were analyzed at every passage as shown on the schematic experimental design (Additional file 1: Figure S1).

### ASC characterization

ASCs were characterized by surface marker expression (immunophenotype) and the ability to differentiate into adipocytes. Immunophenotype was assessed on SVF and at each passage (P0 to P5) using methods previously described [22]. ASCs were induced to differentiate into adipocytes at P5, and adipogenesis was measured using methods previously described [17, 22]. Data and experimental design (Additional file 1: Figure S1) can be found in Additional file 1.

### RNA isolation and quality

ASCs were expanded in FBS or pHPL and RNA was isolated at each passage. At confluence, the cells were dissociated using trypLE and counted. Thereafter, 1 × 10^6^ cells were centrifuged (300*g*) and the resultant pellet was washed using phosphate buffered saline (PBS). RNA was isolated using the RNeasy Minikit (Qiagen, Hilden, Germany) according to the manufacturer’s instructions, and quantified on a NanoDrop® ND 1000 spectrophotometer (Thermo Fisher Scientific, Waltham, MA, USA). RNA purity was assessed at an absorbance optical density (OD) ratio of 260/280 and 260/230. RNA integrity and quality were assessed using a TapeStation® 2200 (Agilent Technologies; Santa Clara, CA, USA) together with RNA ScreenTape® and Sample Buffer kit (Agilent Technologies, Santa Clara, CA, USA) according to the manufacturer’s instructions. Sample read-out was compared to a TapeStation® RNA ladder. RNA that had absorbance OD ratios greater than 2 and RIN values greater than 8 was used for downstream applications.

### Microarray gene expression analysis

Total RNA (100 ng) isolated from ASCs expanded in FBS or pHPL from P0 to P5 was used for first- and second-strand cDNA syntheses, followed by the synthesis and amplification of complementary RNA (cRNA) by in vitro transcription using an Affymetrix GeneChip® WT PLUS Reagent Kit according to the manufacturer’s protocol. Amplified cRNA was purified using magnetic purification beads. Thereafter, 15 μg of purified cRNA was used to synthesize second cycle single-stranded cDNA (ss-cDNA) and subsequently followed by another purification step. Purified ss-cDNA (5.5 μg) was fragmented, labeled, and used to prepare a hybridization cocktail. Hybridization was performed using the Affymetrix GeneChip® Hybridization Wash and Stain Kit according to the manufacturer’s protocol. The hybridization cocktail was hybridized to Affymetrix GeneChip® Human Gene 2.0 ST arrays. Arrays were placed in an Affymetrix GeneChip® Hybridization Oven-645 rotating at 60 rpm at 45 °C for 17 h, after which they were washed and stained in an Affymetrix GeneChip® Fluidics Station-450Dx before being scanned in an Affymetrix GeneChip® Scanner-7G. The output Affymetrix CEL files, which have intensity values for all probes present on the scanned arrays, were used for further analysis. The Robust Multiarray Analysis algorithm [23] in the Affymetrix Expression Console™ was used to perform background correction, summarization, normalization, and the calculation of probe set expression values. Finally, the Affymetrix Transcription Analysis Console™ was used to calculate the fold change of each probe set or transcript cluster identifier number and mapped to the corresponding gene. Only differentially expressed genes (DEGs) that had a fold-change ≥ 2 or ≤ − 2, a *p* value > 0.05, and an FDR > 0.5 were used for downstream analysis. The fold-change of each gene represents the change in gene expression seen between two samples or conditions being compared and is based on the signal measured.

### Functional analysis

The DEGs for the different samples were used for functional analysis to determine significantly enriched pathways and processes using the g:GOSt functional enrichment analysis tool on the g:Profiler web server [24].

## Results

### ASC characterization

pHPL-ASCs had a tighter, smaller elongated shape when compared to FBS-ASCs (Additional file 1: Figure S2). The immunophenotype of FBS-ASCs and pHPL-ASCs was determined at each passage. More than 90% had the expression profile CD44+CD45−CD73+CD90+CD105+, while fewer than 2% were CD31+CD73−CD105−, and this was maintained up to P5 (Additional file 1: Figure S3). FBS-ASCs and pHPL-ASCs both underwent adipogenesis as evidenced by the accumulation of lipid droplets (Additional file 1: Figure S4).

### Gene expression analysis of ASCs expanded in pHPL and FBS

**Fig. 1:**
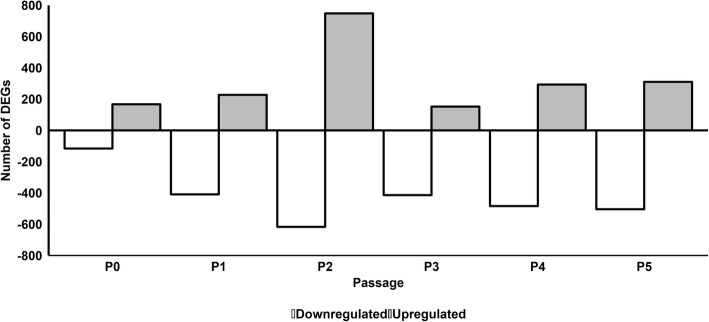
Number of differentially expressed genes in pHPL-ASCs compared to FBS-ASCs at each passage. The gray and white bars represent up- and downregulated genes respectively in pHPL-ASCs when compared to FBS-ASCs at each passage. Volcano plots for these DEGs can be found in Additional file 1: Figure S5)

To compare at the effect of pHPL versus FBS on the transcriptome, we performed a microarray analysis of gene expression on ASCs serially expanded in pHPL or FBS from P0 to P5. We found that 185, 256, 811, 171, 319, and 349 genes were significantly upregulated while 127, 457, 707, 457, 575, and 567 genes were significantly downregulated in ASCs expanded in pHPL (pHPL-ASCs) compared to FBS (FBS-ASCs) at P0, P1, P2, P3, P4, and P5 respectively (Fig. 1; Additional file 1: Figure S5 and Additional file 2).

Functional analysis of the DEGs by gene ontology (GO) classification revealed that genes that were significantly upregulated at the different passages were enriched for certain biological processes (BP), cellular components (CC) and molecular functions (MF). Only the top 5 significant GO terms will be discussed here. From P0 to P5, pHPL-ASCs were enriched for GO terms such as developmental processes, cell cycle processes, cellular proliferation, and extracellular matrix and structure organization. FBS-ASCs were enriched for GO terms such as cell proliferation, adhesion, extracellular matrix and structure organization, cardiovascular and vascular development, structure morphogenesis, and other developmental processes (Table 1; Additional file 3).

**Table 1:** Top 5 enriched GO terms for pHPL-ASCs (upregulated) and FBS-ASCs (downregulated) at each passage (P0–P5). Related to Fig. 1

Gene expression	Domain	P0	P1	P2	P3	P4	P5
Upregulated	BP	Regulation of cell proliferation	Extracellular matrix organization	Cell cycle process	Anatomical structure development	Animal organ development	Multicellular organism development
		Cellular developmental process	Extracellular structure organization	Cell cycle	Multicellular organism development	Multicellular organism development	Extracellular structure organization
		System development	Multicellular organism development	Chromosome organization	System development	System development	Extracellular matrix organization
		Regulation of developmental process	Anatomical structure development	Mitotic cell cycle process	Developmental process	Tissue development	Anatomical structure development
		Multicellular organism development	System development	Mitotic cell cycle	Animal organ development	Anatomical structure development	System development
	CC	Proteinaceous extracellular matrix	Proteinaceous extracellular matrix	Chromosome	Proteinaceous extracellular matrix	Proteinaceous extracellular matrix	Proteinaceous extracellular matrix
		Extracellular matrix	Extracellular matrix	Chromosomal part	Extracellular matrix	Extracellular matrix	Extracellular matrix
		Extracellular region	Extracellular region	Chromosomal region	Extracellular region part	Focal adhesion	Extracellular region part
		Extracellular region part	Extracellular region part	Nuclear lumen	Striated muscle thin filament	Cell-substrate adherens junction	Collagen trimer
		Extracellular space	Extracellular space	Non-membrane-bounded organelle	Muscle thin filament tropomyosin	Cell-substrate junction	Extracellular region
	MF	Glycosaminoglycan binding	mRNA binding involved in posttranscriptional gene silencing	Protein binding	mRNA binding involved in posttranscriptional gene silencing	Oxidoreductase activity, oxidizing metal ions	Transcription factor activity, RNA polymerase ii core promoter proximal region sequence-specific binding
		Ion binding	Collagen binding	Catalytic activity, acting on DNA	mRNA binding	Metalloendopeptidase activity	Metalloendopeptidase activity
		Platelet-derived growth factor-activated receptor activity	Extracellular matrix structural constituent	Carbohydrate derivative binding	Growth factor binding	[heparan sulfate]-glucosamine 3-sulfotransferase 3 activity	Metal ion binding
		Heparin binding	Platelet-derived growth factor receptor binding	Adenyl ribonucleotide binding	Transforming growth factor beta-activated receptor activity	Ionotropic glutamate receptor binding	Cation binding
		Sulfur compound binding	Ion binding	Adenyl nucleotide binding	Oxidoreductase activity, oxidizing metal ions, NAD or NADP as acceptor	Metallopeptidase activity	Xylosyltransferase activity
Downregulated	BP	Cell proliferation	Biological adhesion	Anatomical structure morphogenesis	Anatomical structure morphogenesis	Cell adhesion	Regulation of multicellular organismal process
		Anatomical structure morphogenesis	Cell adhesion	Multicellular organismal process	Developmental process	Biological adhesion	Developmental process
		Circulatory system development	Multicellular organism development	System development	Vasculature development	Anatomical structure morphogenesis	Anatomical structure development
		Extracellular structure organization	Anatomical structure development	Cell adhesion	Cardiovascular system development	Signaling	Multicellular organism development
		Extracellular matrix organization	Anatomical structure morphogenesis	Developmental process	Anatomical structure development	Regulation of multicellular organismal process	Anatomical structure morphogenesis
	CC	Extracellular region part	Extracellular region	Extracellular region part	Extracellular region part	Extracellular region part	Extracellular region part
		Extracellular space	Extracellular region part	Extracellular region	Extracellular region	Extracellular region	Extracellular region
		Extracellular matrix	Cell periphery	Extracellular space	Extracellular space	Extracellular space	Extracellular space
		Extracellular matrix component	Plasma membrane part	Extracellular matrix	Proteinaceous extracellular matrix	Extracellular matrix	Proteinaceous extracellular matrix
		Integral component of plasma membrane	Cell surface	Proteinaceous extracellular matrix	Extracellular matrix	Proteinaceous extracellular matrix	Extracellular matrix
	MF	Insulin-like growth factor binding	Cell adhesion molecule binding	Growth factor binding	Glycosaminoglycan binding	Glycosaminoglycan binding	Glycosaminoglycan binding
		Collagen binding	Glycosaminoglycan binding	Receptor binding	Sulfur compound binding	Heparin binding	Sulfur compound binding
		Protein-lysine 6-oxidase activity	Growth factor binding	Extracellular matrix structural constituent	Heparin binding	Sulfur compound binding	Receptor binding
		Transition metal ion binding	Receptor binding	Glycosaminoglycan binding	Extracellular matrix structural constituent	Extracellular matrix structural constituent	Extracellular matrix structural constituent
		Protein binding	Integrin binding	Insulin-like growth factor binding	Growth factor binding	Growth factor binding	Heparin binding

**Fig. 2:**
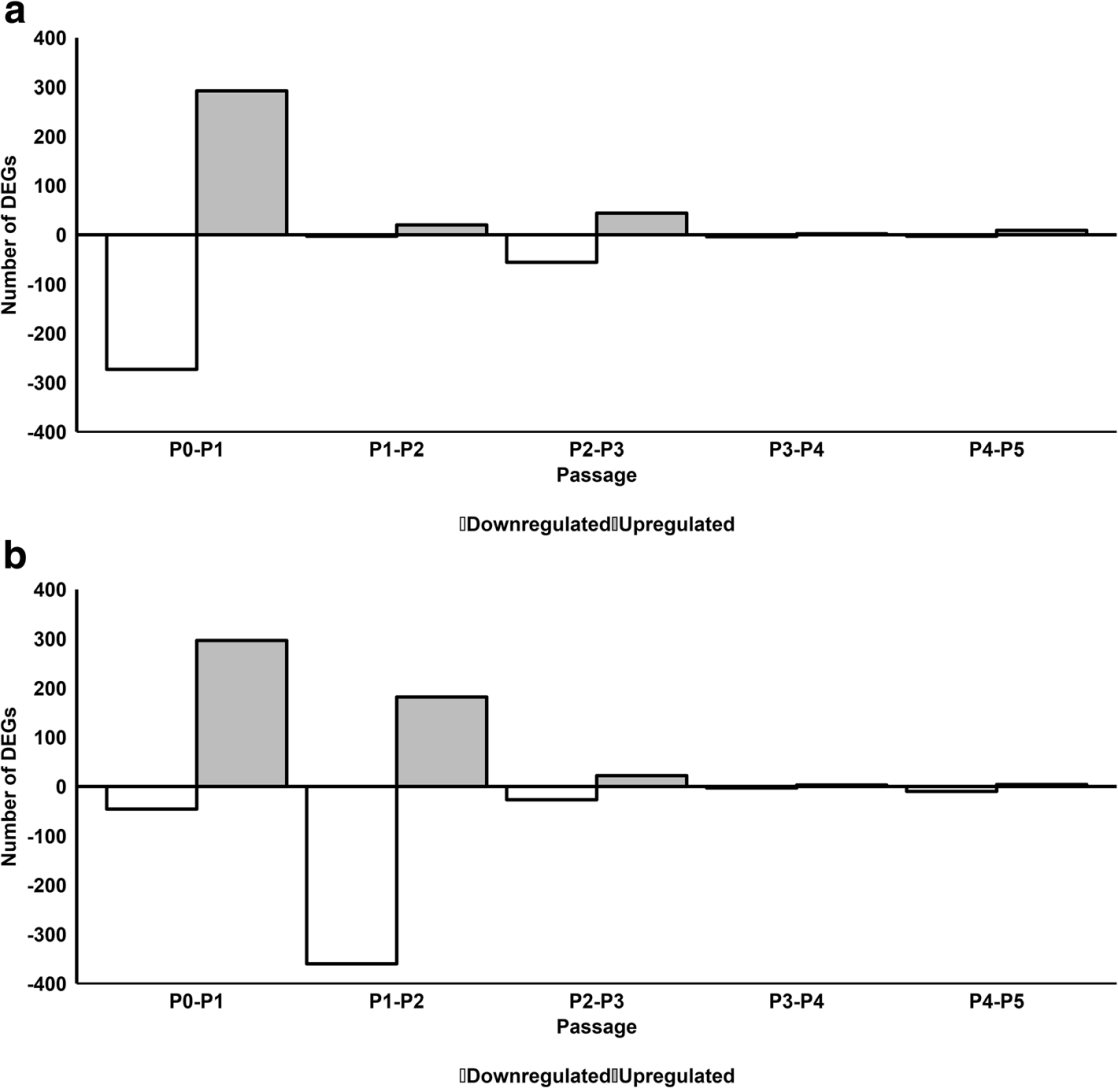
Number of differentially expressed genes during serial passaging of FBS-ASCs (**a**) or pHPL-ASCs (**b**). Gray bars above the horizontal axis are upregulated genes and white bars below the horizontal axis are downregulated genes

We next investigated the effect of serial passaging on gene expression in pHPL-ASCs and FBS-ASCs by comparing gene expression at each passage to that of the previous passage (P1 vs P0, P2 vs P1, P3 vs P2, P4 vs P3, and P5 vs P4). For FBS-ASCs, 292, 20, 44, 2, and 9 genes were significantly upregulated while 273, 3, 56, 4, and 3 genes were significantly downregulated from P0 to P5, respectively (Fig. 2a and Additional file 4). For pHPL-ASCs, 297,182, 22, 3, and 4 genes were significantly upregulated while 46, 360, 27, 3, and 4 genes were significantly downregulated from passages P0 to P5, respectively (Fig. 2b and Additional file 5).

GO classification of upregulated genes in FBS-ASCs revealed they were significantly enriched for cell migration and motility from P0 to P1, while those for P1 to P2 and P2 to P3 were mostly enriched for immunological responses and processes. Genes that were upregulated from P3 to P4 and P4 to P5 were not enriched for any GO terms (Table 2; Additional file 6). Genes that were downregulated from P0 to P1 and P1 to P2 were enriched for system and developmental processes, while those from P2 to P3 were enriched for immune subunit and protein assembly. In contrast, downregulated genes from P3 to P4 and P4 to P5 were not enriched for any GO terms.

**Table 2:** Top 5 enriched GO terms for significantly up- and downregulated DEGs for FBS-ASCs between subsequent passages. Related to Fig. 2a

Gene expression	Domain	P0–P1	P1–P2	P2–P3	P3–P4	P4–P5
Upregulated	BP	Cell migration	Immune system process	Protein-carbohydrate complex subunit organization	–	–
		Immune system process	Immune response	Polysaccharide assembly with MHC class II protein complex	–	–
		Leukocyte migration	Defense response	Protein-carbohydrate complex assembly	–	–
		Localization of cell	Response to stimulus	Antigen processing and presentation of polysaccharide antigen via MHC class II	–	–
		Cell motility	Inflammatory response	MHC class II protein complex assembly	–	–
	CC	Cell surface	Plasma membrane	MHC class II protein complex	–	–
		Plasma membrane	Cell periphery	Lumenal side of endoplasmic reticulum membrane	–	–
		Cell periphery	Plasma membrane part	Integral component of lumenal side of endoplasmic reticulum membrane	–	–
		Integral component of membrane	Intrinsic component of plasma membrane	MHC protein complex	–	–
		Extracellular region	Integral component of plasma membrane	Crlf-clcf1 complex	–	–
	MF	Receptor binding	Receptor binding	MHC class II receptor activity	–	–
		Chemokine activity	Receptor activity	MHC class II protein complex binding	–	–
		Receptor activity	Molecular transducer activity	MHC protein complex binding	–	–
		Cytokine activity	Peptide antigen binding	Peptide antigen binding	–	–
		Molecular transducer activity	Chemokine activity	Leptomycin b binding	–	–
Downregulated	BP	System development	Multicellular organism development	Protein-carbohydrate complex subunit organization	Spliceosomal complex disassembly	–
		Multicellular organism development	System development	Polysaccharide assembly with MHC class II protein complex	Ribonucleoprotein complex disassembly	–
		Developmental process	Anatomical structure development	Protein-carbohydrate complex assembly	–	–
		Anatomical structure development	Developmental process	Antigen processing and presentation of polysaccharide antigen via MHC class II	–	–
		Tissue development	Anatomical structure morphogenesis	MHC class II protein complex assembly	–	–
	CC	Vesicle	Extracellular region part	MHC class II protein complex	U2-type post-mRNA release spliceosomal complex	–
		Extracellular region	Extracellular region	Lumenal side of endoplasmic reticulum membrane	Post-mRNA release spliceosomal complex	–
		Extracellular region part	Extracellular space	Integral component of lumenal side of endoplasmic reticulum membrane	U2-type spliceosomal complex	–
		Extracellular space	Cell periphery	MHC protein complex	–	–
		Cell periphery	Plasma membrane	Crlf-clcf1 complex	–	–
	MF	Glycosaminoglycan binding	Cell adhesion molecule binding	MHC class II receptor activity	–	–
		Cell adhesion molecule binding	Receptor binding	MHC class II protein complex binding	–	–
		Heparin binding	Cadherin binding	MHC protein complex binding	–	–
		Sulfur compound binding	Heparin binding	Peptide antigen binding	–	–
		Fibronectin binding	Growth factor binding	Leptomycin b binding	–	–

For pHPL-ASCs, GO terms significantly enriched for in upregulated genes were immune responses from P0 to P1, regulation of developmental processes and stimulus responses from P1 to P2, RNA binding regulation and transcription factor activity from P2 to P3 and regulation of cardiovascular processes from P3 and P4. Genes that were upregulated from P4 to P5 were not enriched for any GO term (Table 3; Additional file 7). Genes that were downregulated from P1 to P2 were significantly enriched for cell cycle processes, from P2 to P3 for cardiovascular processes, while downregulated genes from P0 to P1, P3 to P4, and P4 to P5 were not enriched for any GO term.

**Table 3:** Top 5 enriched GO terms for significantly up- and downregulated DEGs for pHPL-ASCs between subsequent passages. Related to Fig. 2b

Gene expression	Domain	P0–P1	P1–P2	P2–P3	P3–P4	P4–P5
Upregulated	BP	Immune system process	Regulation of multicellular organismal process	Latent virus replication	Positive regulation of heart rate by epinephrine-norepinephrine	–
		Immune response	Regulation of multicellular organismal development	Regulation of RNA binding transcription factor activity	Positive regulation of heart rate by epinephrine	–
		Inflammatory response	Animal organ morphogenesis	Modulation by host of viral RNA-binding transcription factor activity	Regulation of blood pressure	–
		Defense response	Response to external stimulus	Modulation by host of RNA binding by virus	Positive regulation of stress fiber assembly	–
		Cell surface receptor signaling pathway	Inflammatory response	Regulation of DNA strand elongation	Negative regulation of smooth muscle cell migration	–
	CC	Plasma membrane part	Proteinaceous extracellular matrix	Chloride channel complex	Muscle thin filament tropomyosin	–
		Intrinsic component of plasma membrane	Extracellular matrix	Alpha DNA polymerase:primase complex	Striated muscle thin filament	–
		Integral component of plasma membrane	Extracellular region	Ion channel complex	Sarcoglycan complex	–
		Plasma membrane	Extracellular region part	Transmembrane transporter complex	Bleb	–
		Cell surface	Extracellular space	DNA replication factor a complex	Filamentous actin	–
	MF	Receptor activity	Receptor binding	Chloride channel activity	Prostaglandin-endoperoxide synthase activity	–
		Molecular transducer activity	Integrin binding	Anion channel activity	Actin binding	–
		Signal transducer activity	Calcium ion binding	Chloride transmembrane transporter activity	*N*-Acetylglucosamine-6-sulfatase activity	–
		Signaling receptor activity	Sulfur compound binding	Alkylglycerophosphoethanolamine phosphodiesterase activity	Structural constituent of muscle	–
		Chemokine activity	Scavenger receptor activity	Inorganic anion transmembrane transporter activity	Arylsulfatase activity	–
Downregulated	BP	–	Cell cycle	Positive regulation of heart rate by epinephrine-norepinephrine	–	–
		–	Cell cycle process	Positive regulation of heart rate by epinephrine	–	–
		–	Chromosome organization	Regulation of blood pressure	–	–
		–	Mitotic cell cycle	Positive regulation of stress fiber assembly	–	–
		–	Mitotic cell cycle process	Negative regulation of smooth muscle cell migration	–	–
	CC	–	Chromosome	Muscle thin filament tropomyosin	–	–
		–	Chromosomal part	Striated muscle thin filament	–	–
		–	Chromosomal region	Sarcoglycan complex	–	–
		–	Intracellular non-membrane-bounded organelle	Bleb	–	–
		–	Non-membrane-bounded organelle	Filamentous actin	–	–
	MF	–	Protein binding	Prostaglandin-endoperoxide synthase activity	–	–
		–	Catalytic activity, acting on DNA	Actin binding	–	–
		–	Adenyl ribonucleotide binding	*N*-Acetylglucosamine-6-sulfatase activity	–	–
		–	ATP binding	Structural constituent of muscle	–	–
		–	Adenyl nucleotide binding	Arylsulfatase activity	–	–

**Fig. 3:**
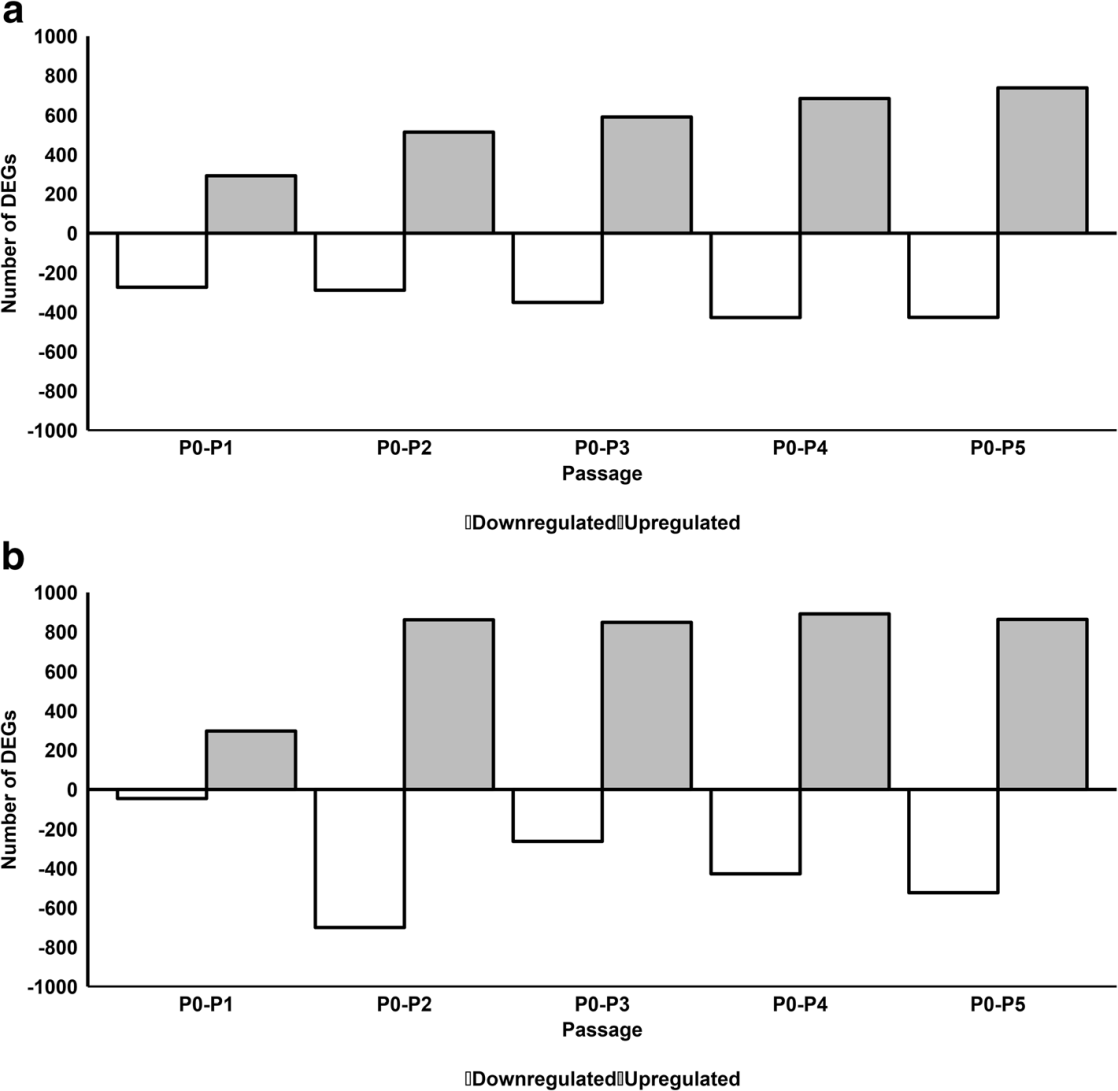
Number of differentially expressed genes when compared to P0 in FBS-ASCs (**a**) or pHPL-ASCs (**b**). Gray bars above the horizontal axis are upregulated genes and white bars below the horizontal axis are downregulated genes

We next undertook to evaluate the extent to which the ASC transcriptome at each passage (P1 through to P5) differs from its original state (SVF) at P0 when expanded in either FBS or pHPL, and to functionally characterize such changes using GO classification. This was done by comparing gene expression at each passage (P1 to P5) to that of the “original” seeded ASCs (SVF) at P0. For FBS-ASCs, 292, 514, 591, 685, and 737 genes were significantly upregulated while 273, 288, 350, 427, and 426 genes were significantly downregulated from P1 to P5 (Fig. 3a and Additional file 8). For pHPL-ASCs, 297, 861, 848, 891, and 863 genes were significantly upregulated while 46, 700, 262, 427, and 523 genes were significantly downregulated from passage P1 to P5 (Fig. 3b and Additional file 9).

GO terms significantly enriched for in upregulated genes at each passage (P1 to P5) when compared to P0 in FBS-ASCs (Table 4; Additional file 10) or pHPL-ASCs (Table 5; Additional file 11) were specific to immune responses and processes. GO terms specific to developmental processes were enriched for in the downregulated genes in FBS-ASCs at each passage (P1 to P5) when compared to P0 (Table 4; Additional file 10). For pHPL-ASCs, downregulated genes at P1 were not enriched for any GO term, while those of all the subsequent passages (P2 to P5) were enriched for cell cycle processes and developmental processes.

**Table 4:** Top 5 enriched GO terms for significantly up- and downregulated DEGs for FBS-ASCs between P0 and subsequent passages. Related to Fig. 3a

Gene expression	Domain	P0–P1	P0–P2	P0–P3	P0–P4	P0–P5
Upregulated	BP	Cell migration	Immune system process	Immune system process	Immune system process	Immune system process
		Immune system process	Immune response	Defense response	Immune response	Immune response
		Leukocyte migration	Defense response	Immune response	Cell surface receptor signaling pathway	Defense response
		Localization of cell	Regulation of immune system process	Inflammatory response	Response to stimulus	Response to stimulus
		Cell motility	Cell surface receptor signaling pathway	Response to stimulus	Defense response	Cell surface receptor signaling pathway
	CC	Cell surface	Plasma membrane	Plasma membrane	Plasma membrane part	Intrinsic component of plasma membrane
		Plasma membrane	Cell periphery	Cell periphery	Intrinsic component of plasma membrane	Plasma membrane
		Cell periphery	Plasma membrane part	Plasma membrane part	Integral component of plasma membrane	Plasma membrane part
		Integral component of membrane	Intrinsic component of plasma membrane	Intrinsic component of plasma membrane	Plasma membrane	Integral component of plasma membrane
		Extracellular region	Integral component of plasma membrane	Integral component of plasma membrane	Cell periphery	Cell periphery
	MF	Receptor binding	Receptor activity	Receptor activity	Receptor activity	Receptor binding
		Chemokine activity	Molecular transducer activity	Molecular transducer activity	Molecular transducer activity	Receptor activity
		Receptor activity	Receptor binding	Chemokine activity	Receptor binding	Molecular transducer activity
		Cytokine activity	Chemokine activity	Receptor binding	Peptide antigen binding	Peptide antigen binding
		Molecular transducer activity	Chemokine receptor binding	Signaling receptor activity	Chemokine activity	Chemokine activity
Downregulated	BP	System development	Anatomical structure development	System development	Anatomical structure development	System development
		Multicellular organism development	Multicellular organism development	Multicellular organism development	Developmental process	Developmental process
		Developmental process	Anatomical structure morphogenesis	Cell adhesion	Multicellular organism development	Multicellular organism development
		Anatomical structure development	Nervous system development	Biological adhesion	System development	Anatomical structure development
		Tissue development	System development	Developmental process	Anatomical structure morphogenesis	Anatomical structure morphogenesis
	CC	Vesicle	Extracellular region	Extracellular region part	Extracellular region part	Extracellular region part
		Extracellular region	Extracellular region part	Proteinaceous extracellular matrix	Extracellular region	Cell periphery
		Extracellular region part	Cell periphery	Extracellular region	Proteinaceous extracellular matrix	Proteinaceous extracellular matrix
		Extracellular space	Extracellular space	Extracellular matrix	Extracellular matrix	Extracellular region
		Cell periphery	Extracellular matrix	Extracellular space	Extracellular space	Plasma membrane
	MF	Glycosaminoglycan binding	Cell adhesion molecule binding	Glycosaminoglycan binding	Sulfur dioxygenase activity	Cell adhesion molecule binding
		Cell adhesion molecule binding	Neuropilin binding	Heparin binding	Glycosaminoglycan binding	Cadherin binding
		Heparin binding	Transporter activity	Receptor binding	Heparin binding	Receptor binding
		Sulfur compound binding	Cadherin binding	Sulfur compound binding	Cell adhesion molecule binding	Neuropilin binding
		Fibronectin binding	Protein tyrosine kinase activator activity	Ion binding	Cadherin binding	Actin binding

**Table 5:** Top 5 enriched GO terms for significantly up- and downregulated DEGs for pHPL-ASCs between P0 and subsequent passages. Related to Fig. 3b

Gene expression	Domain	P0–P1	P0–P2	P0–P3	P0–P4	P0–P5
Upregulated	BP	Immune system process	Immune system process	Immune system process	Immune system process	Immune system process
		Immune response	Immune response	Immune response	Immune response	Immune response
		Inflammatory response	Inflammatory response	Response to external stimulus	Response to external stimulus	Response to external stimulus
		Defense response	Defense response	Defense response	Defense response	Defense response
		Cell surface receptor signaling pathway	Cellular response to chemical stimulus	Cellular response to chemical stimulus	Inflammatory response	Cellular response to chemical stimulus
	CC	Plasma membrane part	Extracellular region	Extracellular region	Extracellular region	Extracellular region
		Intrinsic component of plasma membrane	Extracellular region part	Plasma membrane	Intrinsic component of plasma membrane	Extracellular region part
		Integral component of plasma membrane	Plasma membrane part	Cell periphery	Integral component of plasma membrane	Plasma membrane part
		Plasma membrane	Intrinsic component of plasma membrane	Intrinsic component of plasma membrane	Plasma membrane part	Extracellular space
		Cell surface	Extracellular space	Plasma membrane part	Extracellular region part	Plasma membrane
	MF	Receptor activity	Receptor activity	Receptor activity	Receptor activity	Receptor binding
		Molecular transducer activity	Receptor binding	Glycosaminoglycan binding	Molecular transducer activity	Receptor activity
		Signal transducer activity	Molecular transducer activity	Molecular transducer activity	Receptor binding	Molecular transducer activity
		Signaling receptor activity	Glycosaminoglycan binding	Receptor binding	Glycosaminoglycan binding	Glycosaminoglycan binding
		Chemokine activity	Cytokine binding	Sulfur compound binding	Peptide binding	Signal transducer activity
Downregulated	BP	–	Cell cycle process	Anatomical structure morphogenesis	Cell cycle process	Cell cycle process
		–	Cell cycle	Developmental process	Cell division	Cell division
		–	Mitotic cell cycle	Anatomical structure development	Anatomical structure morphogenesis	Chromosome segregation
		–	Mitotic cell cycle process	System development	Tissue development	Nuclear chromosome segregation
		–	Chromosome organization	Tissue development	Mitotic cell cycle process	Sister chromatid segregation
	CC	–	Chromosome	Plasma membrane raft	Spindle	Chromosome, centromeric region
		–	Chromosomal part	Postsynapse	Condensed chromosome outer kinetochore	Condensed chromosome, centromeric region
		–	Chromosomal region	Caveola	Cytoskeleton	Spindle
		–	Chromosome, centromeric region	Z disc	Mitotic spindle	Kinetochore
		–	Nuclear lumen	Postsynaptic density	Condensed chromosome kinetochore	Condensed chromosome kinetochore
	MF	–	Catalytic activity, acting on DNA	2-Aminoadipate transaminase activity	Microtubule binding	ATP binding
		–	Protein binding	Protein-lysine 6-oxidase activity	Cell adhesion molecule binding	Adenyl ribonucleotide binding
		–	DNA-dependent ATPase activity	Binding	Tubulin binding	Adenyl nucleotide binding
		–	Chromatin binding	Kynurenine aminotransferase activity	Cytoskeletal protein binding	Microtubule binding
		–	ATP binding	Kynurenine-oxoglutarate transaminase activity	Kinase activity	Cell adhesion molecule binding

We observed during serial passaging that the ASC transcriptomic profile stabilizes (minimal change in DEGs between adjacent passage numbers) from P2 for FBS (Fig. 2a) and P3 for pHPL (Fig. 2b). This could mean that ASC cultures are more homogenous from P2 to P5 and from P3 to P5 when expanded in FBS and pHPL respectively.

From the list of DEGs obtained at each passage (P1 to P5) when compared to P0 for both the FBS- and pHPL-ASCs (Additional files 8 and 9), we observed that ASCs showed gene expression signatures that were unique at each passage (P1 to P5) which was independent of the medium supplementation (FBS or pHPL) used during in vitro expansion (Additional file 12). This unique passage-specific gene expression profile constitutes the DEGs that were common to both pHPL and FBS at each passage number. Equally, if the passage-specific gene expression profile (DEGs common to both FBS- and pHPL-ASCs at each passage) is excluded at each passage number, the remaining DEGs represent unique FBS-ASC and pHPL-ASC passage-specific gene expression profiles (Additional file 12).

Furthermore, by considering the unique FBS-ASC passage-specific gene expression profile at all passages (P1 to P5), there were 37 (AC007879.7, ADAMTS4, ADAMTS9, ALOX5, CCL11, CCL4, CHST1, CLEC5A, COL6A3, CRISPLD2, CTHRC1, DCHS1, DOCK4, FIBIN, GALNT15, HEPH, HEY2, IL3RA, MCTP1, MMP1, NPAS2, PALMD, PIM1, PLAU, PLAUR, PREX1, RGS1, SNAI1, SRPX2, SYTL2, TDO2, TEAD2, THEMIS2, TNC, TNFAIP8L1, WAS, and WSB1) and 81 (ADAMTS1, AHNAK2, ALDH7A1, ANKRD1, ANKRD37, ARHGAP29, ARSK, ASAP2, ATP10D, ATP8B1, BAMBI, BCHE, BMP4, BST1, C11orf87, CCND1, CDH6, COMP, COX7A1, DEPTOR, FAM155A, FAM180A, FAM65B, FGF9, GLRX, GPR133, GPRC5A, GREM1, GREM2, HAPLN1, HSPB6, IGFBP5, IGFBP6, IL1RAPL2, KCTD16, KRT14, KRT18, LIMCH1, LURAP1L, MANSC1, MKX, MYOZ2, NCKAP5, NDFIP2, NIPAL3, NLRP10, NOV, NPR3, NR3C2, NRK, NTRK3, OXTR, PAPSS2, PDE1A, PDE1C, PI16, PKP2, PPL, RCAN2, RGS7BP, RHOJ, ROR1, RP11-553 K8.5, RP11-760H22.2, RP11-818F20.5, SAMD12, SBSPON, SDPR, SEMA5A, SLC1A1, SMURF2, STS, SYPL2, TIAM2, TINAGL1, TMEM19, TNFRSF11B, USP53, VEPH1, WEE1, and WNT2) genes that were consistently up- or downregulated respectively at all passages (Additional file 13). This represents the set of genes that were differentially expressed in ASCs as a result of them being expanded in FBS irrespective of the cell passage number. This could be reflective of an FBS-specific effect on the ASC transcriptome (FBS-ASC-specific gene expression profile). Similarly, by looking at the unique pHPL-ASC passage-specific gene expression profile at all passages (P1 to P5), there were 32 (A2M, ABLIM1, ADAMTS1, ADCYAP1R1, C10orf10, CHI3L1, EVI2B, F13A1, FAM65B, FST, GALNT12, HLA-QA1, HLA-DQA2, IL18, IL33, JAG1, MGP, MIR548I2, MT1G, MYCBP2, NTRK2, PCDHB16, PCSK1, PRELP, PRG4, RARRES1, ROR1-AS1, SFRP4, SMPDL3A, THBD, TPRG1, and ZNF727P) and 11 (CDK15, CTHRC1, EHD3, MBOAT2, MIR199A2, MIR503, MIR503HG, NT5DC2, PALLD, PPP2R3A, and RP11-08B5.2) genes that were consistently up- or downregulated respectively at all passages (Additional file 13). This represents the set of genes that are differentially expressed in ASCs as a result of them being expanded in pHPL, irrespective of the cell passage number. This could be reflective of a pHPL-specific effect on the ASC transcriptome (pHPL-ASC-specific gene expression profile).

In total therefore, there were 118 DEGs that constituted the FBS-ASC-specific gene expression profile, which is almost 3 times more than the 43 DEGs of the pHPL-ASC-specific gene expression profile (Additional file 14). Functional analysis of the pHPL-ASC-specific gene expression signature by GO classification showed that neither up- nor downregulated genes were enriched for any biological process, while the FBS-ASC-specific gene expression signature showed upregulated genes that were significantly enriched for cell migration and cell movement processes, while the downregulated genes were significantly enriched for the regulation of cell communication, signal transduction and cell proliferation processes.

Since the passage-specific gene expression profile consists of the common genes expressed by both FBS- and pHPL-ASCs at each passage, the genes that are common to all these passage-specific profiles will then constitute an ASC gene expression profile that is not affected by medium supplementation or cell passage number. There are 69 upregulated genes (AIF1, APCDD1, APLN, APOC1, AQP9, BCL6B, C1orf162, C5AR1, CADM3, CCDC102B, CCR1, CD14, CD37, CD53, CD93, CDH5, CLEC7A, CLIC6, CPM, CSF1R, CSF2RA, CXCL16, CXCR4, CXorf36, ECSCR, ELMO1, ENPEP, FCER1G, FPR3, GMFG, GUCY1A3, HPGDS, IL18R1, ITGAM, ITGAX, KDR, KYNU, LAPTM5, LCP1, LCP2, LRRC25, LYVE1, MERTK, MGAT4A, NCF2, NCKAP1L, NOTCH3, OLFM2, PAG1, PECAM1, PILRA, PLTP, PLVAP, POM121L9P, PPBP, RAMP2, RNASE6, SCG2, SLC11A1, SLC16A10, SPARCL1, SPP1, TM4SF18, TMEM176B, TNFRSF1B, TREM1, TREM2, TYROBP, and VSIG4) and 5 downregulated genes (F2RL2, FGF5, GALNT5, RAB3B, and SLC9A7) that constitute this subset of genes that were consistently differentially expressed from P1 through to P5. This set of genes therefore represents a unique in vitro ASC transcriptome profile that was neither affected by medium supplementation nor cell passage number (Additional file 14). GO classification of these genes revealed that they are significantly enriched for normal cellular processes like response to stimulus and stress, defense, and inflammatory responses and vesicle-mediated transport.

## Discussion

Adipose-derived stromal cells (ASCs) are being assessed for their safety and efficacy in numerous clinical trials [6, 14, 25]. Traditionally, these cells are expanded in medium containing FBS, which is known to have several disadvantages such as the transmission of zoonotic diseases and the stimulation of immune reactions in patients [26, 27]. This has been circumvented by changing from animal products to either clinical-grade, GMP-compliant, or human alternative products [28]. One such change has been to supplement culture medium with either serum-free media or human blood components. The use of different medium supplements has been well documented and all show comparable immunophenotypic profiles and differentiation capacities while having marked differences in proliferation capacity [6]. The advantage of pHPL over these alternatives lies largely in the ability to pool platelets from multiple donors. Furthermore, it has been shown that ASCs expanded in pHPL retain their immunophenotypic characteristics and their ability to differentiate into bone, cartilage and fat [2, 6, 16]. One of the biggest advantages of using pHPL for ASC expansion is the marked increase in proliferation, which in turn makes the time required for expansion to therapeutic numbers considerably shorter [12, 22]. However, not much is known about the effect of pHPL has on the transcriptome, proteome, and secretome of these cells, which may impact on the outcome of clinical trials. This study has made use of microarray technology to examine the effect of pHPL on the ASC transcriptome during serial expansion in vitro, by comparing gene expression patterns in cells serially expanded in FBS or pHPL from P0 to P5.

Overall, the transcriptome of ASCs expanded in pHPL or FBS was most different at P2, the point at which the maximum number of genes were differentially expressed (811 and 707, respectively; Fig. 1). Most genes that were upregulated in pHPL-ASC were significantly enriched for biological process such as cell cycle, cell division, and proliferation. This supports a previous study by Glovinski et al., in which changes in the expression of genes involved in cell proliferation and development were observed for ASCs expanded in pHPL [12]. This likewise confirms findings from other studies which have shown an increase in ASC proliferation in pHPL [16, 29]. For ASCs expanded in FBS, our findings are consistent with the observation that numerous genes involved in extracellular matrix formation are upregulated [30, 31].

It is well documented that ASCs are a heterogeneous population as revealed by differences in transcriptome, proteome, and secretome between subpopulations within the ASC mixture [32–34]. The initial subset of adherent cells seeded in culture (P0) is a heterogeneous population; after passaging and prolonged expansion, the population becomes more homogenous [35]. Work performed by several groups has shown that the heterogeneity of ASCs during the expansion process remains between subpopulations and between individual cells within the same subpopulation [32, 36, 37]. Furthermore, it has been established that serial passaging affects ASC gene expression profiles [29]. Global gene expression profiles could therefore be used as a tool to study ASC heterogeneity at different passages. The more homogenous the cultures are at different passages, the fewer the number of DEGs will be between them.

We have investigated the effect of serial passaging on the ASC transcriptome by comparing FBS-ASC and pHPL-ASC cultures at each passage to those of the previous passage. We observed that the transcriptome was relatively stable from P2 to P5 for cells expanded in FBS and from P3 to P5 for cells expanded in pHPL as is evident from the relatively low number of DEGs obtained between these passages. This suggests the ASC cultures become homogenous at the transcriptome level earlier in FBS (P2) than in pHPL (P3). Interestingly, the genes upregulated significantly in FBS-ASCs were enriched for biological processes involved in immune and inflammatory responses. These findings are similar to those reported by Kim et al., where genes involved in inflammatory and immune responses, and cell migration and homing [19], were upregulated in ASCs expanded in FBS. They further postulated that the upregulation of these genes was due to the high cell density at the time of cell harvesting and could be the reason why FBS-expanded ASCs might be effective in treating graft-vs-host disease and damaged tissues. On the other hand, human clinical trials that have made use of ASCs expanded in FBS have reported adverse immune responses in patients after administration [2, 6–8, 38]. This could be due to the upregulation of these inflammatory and immune response genes. Genes that were downregulated in ASCs expanded in FBS at early passages (P0 to P1) were enriched for biological processes involving tissue development. Other studies have reported similar findings [30] which may explain why differentiation into adipocytes is reduced at later passages [18, 39]. Surprisingly, genes that were upregulated in pHPL-ASCs at earlier passages were also enriched for immune and inflammatory response processes. This could be due to the presence of immune cells in the early passages and may not be related to the serum used. To further explore the possible presence of immune cells in early passages (P0), we compared each passage (P1 to P5) to P0. It was observed that upregulated genes were significantly enriched for immune and inflammatory responses irrespective of the supplementation used, while the downregulated genes were enriched for tissue developmental and cell cycle and division processes. To assess serum-specific transcriptional changes (where genes were differentially expressed based on the serum supplementation used), we normalized gene expression at all other passages to the passage at which the transcriptome stabilizes (P2 for FBS-ASCs and P3 for pHPL-ASCs). For FBS-ASCs, the upregulated genes were enriched for immune and inflammatory responses; this supports the findings obtained when we compared each passage to the previous passage and each passage to P0. This may suggest that FBS-ASCs express genes that are involved in immune reactions; however, the functional implications of this in clinical or in vivo settings will need to be explored further. Genes that were downregulated in FBS-ASCs were enriched for structure, organ, and tissue developmental processes suggesting that ASCs have greater differentiation potential at earlier passages such as P2. For ASCs expanded in pHPL, upregulated genes were enriched for DNA and RNA regulation processes, BMP pathway signaling, and cell cycle and cell division processes. These findings suggest that proliferation may not decrease with increased passaging as indicated by Shahdadfar et al. [15] and could provide therapeutic numbers more readily than other human alternatives and FBS*.*

ASCs showed passage and serum-specific gene expression profiles. The passage-specific gene expression profile which is comprised of the DEGs that are common to both pHPL and FBS at each passage might reflect the in vitro serial passaging effect on the ASC transcriptome. The serum-specific gene expression signature at each passage (P1 to P5) may be reflective of the FBS or pHPL effect on the ASC transcriptome at that time period in culture (passage number) during the serial expansion process.

There were 118 and 43 genes that were differentially expressed in ASCs throughout the serial expansion process in FBS and pHPL respectively. This might indicate an ASC transcriptome profile that is specific to the medium supplementation (FBS or pHPL) used during cell expansion, irrespective of passage number. Thus, a serum-specific signature could potentially be used to identify the medium supplement (FBS or pHPL) in which the cells were previously expanded. This in turn could inform decision making in terms of the downstream clinical/research applications of these cells. There were fewer DEGs obtained for the pHPL-ASC-specific gene expression signature (43 genes), which is 1/3 the number of DEGS observed in FBS-ASCs (118 genes). The pHPL-ASC-specific gene expression signature was not enriched for any biological processes unlike the FBS-ASC-specific expression signature. This could mean that pHPL has no significant effect on the ASC transcriptome during in vitro serial passaging and suggests that pHPL might be a better medium supplement than FBS for in vitro cell expansion. Furthermore, downregulated genes in the FBS-ASC-specific gene expression signature were enriched for cell proliferation processes. This supports the observation that ASCs grow slower in FBS when compared to cell-expanded pHPL.

Finally, we observed that ASCs have a unique in vitro transcriptome profile, which is independent of cell passage number and/or medium supplementation. This consists of a set of genes that are always expressed by ASCs in vitro at any given time in culture during the expansion process (P1 to P5). Interestingly, some of the genes constituting this unique in vitro ASC transcriptome have previously been reported to be expressed by ASCs. Thus, ASCs express CXCR4 and CCR1 at both protein and mRNA levels [40]. PECAM-1 has been reported to be expressed by ASCs especially during early passages [41, 42]. ITGAM is another gene shown to be expressed by ASCs at low levels up to P3, and exhibits greater than 70% isoform switching between experimental conditions [43]. CD53 and TREM1 have been reported recently as novel marker genes expressed by adipogenic progenitor preadipocyte cells and BCL6B by osteochondrogenic progenitor preadipocyte cells from mouse bone marrow [44]. Furthermore, a novel subpopulation of human adipose tissue-resident macrophages (ATMs) located in the interstitial spaces between adipocytes has been shown to express CD14, which upon culturing to P3 is lost, at which point the cells display an expression profile which is similar to ASCs [45]. Therefore, the expression of CD14 by ASCs in this study suggests the presence of a heterogeneous population of ASCs that contains this novel subpopulation of ATMs which persisted beyond P3 in culture.

The entire process of obtaining a product for clinical purposes should adhere to the GMP guidelines. The use pHPL for the expansion of ASCs in vitro is one of many steps required. In this study, we made use of defined, clinical-grade reagents and the expansion of the ASCs was performed under sterile conditions. Isolation and expansion of ASCs in a closed system to further reduce the risk of contamination would provide a robust clinical GMP-complaint process.

## Conclusion

This study highlights differences in the transcriptome of ASCs expanded in pHPL versus FBS, which could be used to guide their application in the clinical setting. ASCs expanded in FBS were enriched for immune and inflammatory responses, whereas ASCs expanded in pHPL were enriched for cell cycle, proliferation, and cell division. Our findings suggest that the differentiation capacity of ASCs is likely to be greater at earlier passages and that ASCs expanded in pHPL are likely to retain their proliferative capacity during prolonged expansion. These findings also suggest pHPL may be a superior supplement for expanding ASCs to therapeutic numbers without influencing the expression of genes involved in differentiation of specific developmental processes. Furthermore, we found that even though ASCs expanded in pHPL had a greater proliferation capacity, they were not enriched for genes specific to transformation. While these findings provide novel insights into potential markers for ASCs, some of the individual genes and groups of genes mentioned in this study need to be further investigated. Finally, to further compliment these findings, we believe that the proteome and the secretome of ASCs expanded in pHPL or FBS should also be studied.

## Additional files

Additional file 1: ASC characterization methods and results and volcano plots of DEGs between ASCs expanded in FBS and pHPL. ASC morphology, immunophenotype and differentiation, results and materials and methods, and volcano plots of DEGs between ASCs expanded in FBS and pHPL. (DOCX 2659 kb)

Additional file 2: Up- and downregulated gene list for ASCs serially expanded in FBS and pHPL. Complete list of up- and downregulated genes for ASCs serially expanded in pHPL or FBS (P0 – P5). This data relates to Fig. 1. (XLSX 193 kb)

Additional file 3 Gene ontology terms for ASCs serially expanded in FBS and pHPL. Complete list of enriched GO terms for ASCs serially expanded in pHPL or FBS (P0 – P5). This data relates to Table 1. (XLSX 1017 kb)

Additional file 4: Up- and downregulated gene list for ASCs expanded in FBS between subsequent passages. Complete list of up- and downregulated genes for FBS-ASCs between subsequent passages (P0 - P1, P1 - P2, P2 - P3, P3 - P4, P4 - P5). This data relates to Fig. 2a. (XLSX 88 kb)

Additional file 5: Up- and downregulated gene list for ASCs expanded in pHPL between subsequent passages. Complete list of up- and downregulated genes for pHPL-ASCs between subsequent passages (P0 - P1, P1 - P2, P2 - P3, P3 - P4, P4 - P5). This data relates to Fig. 2b. (XLSX 58 kb)

Additional file 6 Gene ontology terms for ASCs expanded in FBS between subsequent passages. Complete list of enriched GO terms for FBS-ASCs between subsequent passages (P0 - P1, P1 - P2, P2 - P3, P3 - P4, P4 - P5). This data relates to Table 2. (XLSX 516 kb)

Additional file 7: Gene ontology terms for ASCs expanded in pHPL between subsequent passages. Complete list of enriched GO terms for pHPL-ASCs between subsequent passages (P0 - P1, P1 - P2, P2 - P3, P3 - P4, P4 - P5). This data relates to Table 3. (XLSX 349 kb)

Additional file 8: Up- and downregulated gene list for ASCs expanded in FBS between P0 and subsequent passages. Complete list of up- and downregulated genes for FBS-ASCs between P0 and subsequent passages (P0 - P1, P0 - P2, P0 - P3, P0 - P4, P0 - P5). This data relates to Fig. 3a. (XLSX 166 kb)

Additional file 9: Up- and downregulated gene list for ASCs expanded in pHPL between P0 and subsequent passages. Complete list of up- and downregulated genes for pHPL-ASCs between P0 and subsequent passages (P0 - P1, P0 - P2, P0 - P3, P0 - P4, P0 - P5). This data relates to Fig. 3b. (XLSX 206 kb)

Additional file 10: Gene ontology terms for ASCs expanded in FBS between P0 and subsequent passages. Complete list of enriched GO terms for FBS-ASCs between P0 and subsequent passages (P0 - P1, P0 - P2, P0 - P3, P0 - P4, P0 - P5). This data relates to Table 4. (XLSX 1145 kb)

Additional file 11: Gene ontology terms for ASCs expanded in pHPL between P0 and subsequent passages. Complete list of enriched GO terms for pHPL-ASCs between P0 and subsequent passages (P0 - P1, P0 - P2, P0 - P3, P0 - P4, P0 - P5). This data relates to Table 5. (XLSX 1289 kb)

Additional file 12: FBS and pHPL-ASC passage specific gene expression profile. A complete list of genes comprising the FBS and pHPL-ASC passage specific gene expression profile. (XLSX 89 kb)

Additional file 13: FBS and pHPL-ASC medium supplementation specific gene expression profile. A complete list of genes comprising the FBS and pHPL-ASC medium supplementation specific gene expression profile. (XLSX 67 kb)

Additional file 14: ASC gene signature irrespective of cell passage number and/or media supplement used. A complete list of genes comprising the ASC gene signature irrespective of cell passage number and/or media supplement used. (XLSX 20 kb)

## Data Availability

The datasets supporting the conclusions of this article are included within the article (and its additional files). The microarray data files of this study will be deposited in NCBI GEO (Gene Expression Omnibus).

## Abbreviations

ASC: Adipose-derived stromal cell
BP: Biological processes
CC: Cellular components
cRNA: Complementary RNA
DEGs: Differentially expressed genes
DNA: Deoxyribose nucleic acid
FBS: Fetal bovine serum
FS: Forward Scatter
GMP: Good manufacturing practices/processes
GO: Gene ontology
IFATS: International Federation of Adipose Therapeutics and Sciences
ISCT: Mesenchymal and Tissue Stem Cell Committee of the International Society for Cellular Therapy
MI: Molecular function
OD: Optical density
P: Passage
p/s: Penicillin/streptomycin
PBS: Phosphate buffered saline
pHPL: Pooled human platelet lysate
ss-cDNA: Single-stranded cDNA
SVF: Stromal vascular fraction
α-MEM: Modified Eagle’s medium - alpha
