# Supplementary material for: The effect of medium supplementation and serial passaging on the transcriptome of human adipose-derived stromal cells expanded in vitro
Source: Stem Cell Res Ther. 2019 Aug 14;10:253. doi: 10.1186/s13287-019-1370-2 (PMC6694630; doi:10.1186/s13287-019-1370-2)
Supplement: Supplementary file 1 — ASC characterization methods and results and volcano plots of DEGs between ASCs expanded in FBS and pHPL. ASC morphology, immunophenotype and differentiation, results and materials and methods, and volcano plots of DEGs between ASCs expanded in FBS and pHPL. (DOCX 2659 kb) [file 13287_2019_1370_MOESM1_ESM.docx]

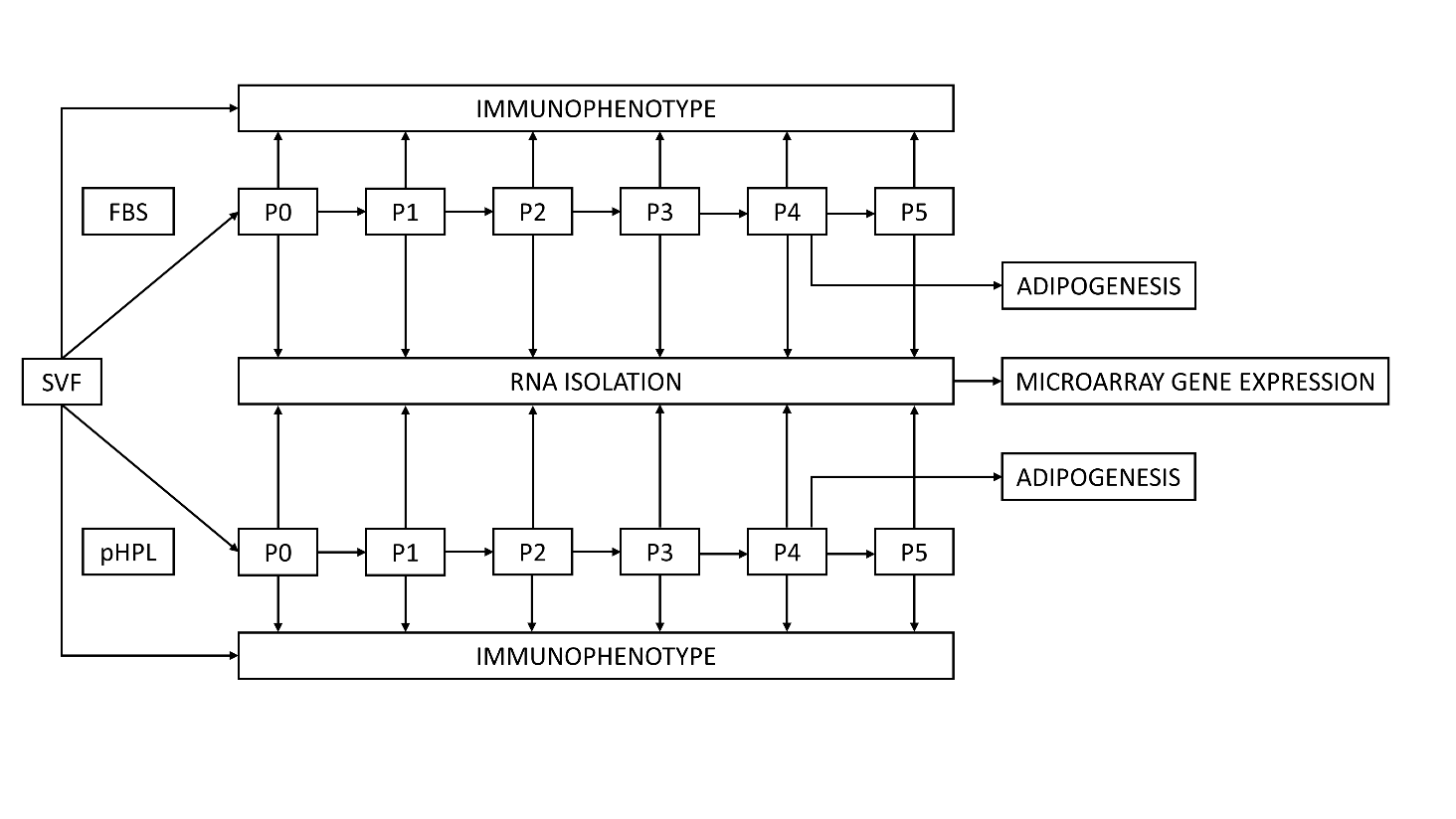


**Figure 1: Experimental design and layout**. SVF was isolated from of five volunteer donors and plated (P0) in either pHPL or FBS supplemented medium. At confluence, cells were dissociated, immunophenotyped and plated for further expansion. ASCs were maintained and expanded for 5 passages. At P4, ASCs were dissociated and plated for adipogenic induction at P5. At each passage, 1 x 10^6^ ASCs were used for RNA isolation. RNA integrity and quality were tested and only RNA with a RIN value greater than 8 and absorption ODs greater than 2 was used for downstream microarray gene expression analysis.

# ASC Characterization

## ASC cumulative population doubling

When the ASCs reached 70 to 80% confluence, the samples were washed twice with 4 mL PBS containing 2% (v/v) p/s. The adherent cells were then detached from the surface of the culture flasks using 3 mL tryPLE (Life Technologies™, New York, USA) and incubated at 37˚C at 5% CO_2_. After 7 to 10 minutes, the flasks were checked under a microscope to ensure that no residual cells were attached. The tryPLE was neutralised by adding 3 mL FBS supplemented medium to the cell suspension in the culture flasks. The cell suspension was then transferred to a 15 mL conical tube. The flasks were washed twice with 4 mL PBS containing 2% (v/v) p/s and transferred to the same 15 mL tube. The cell suspensions were centrifuged at 184 x *g* for 5 minutes, the supernatant was aspirated and discarded, and the cells resuspended in 2 mL PBS containing 2% (v/v) p/s. The cells were prepared for counting by adding 100 µL cell suspension, 100 µL Flow-Count™ fluorospheres and 1 mL PBS to a flow tube. The cells were then counted on a Gallios flow cytometer. The population doubling level (PDL) was calculated using the formula *PDL* = 3.32 (log N_H_ - log N_I_) + X, where *N_H_* = the harvested cell number, *N_I_* = the seeded cell number, and *X* = the doubling level used to initiate the subculture being quantitated.

The ASCs expanded in pHPL had a higher PDL than the ASCs expanded in FBS as seen in table 1.

**Table 1: Population doubling level of ASCs expanded in FBS and pHPL**

|  | **P0** | **P1** | **P2** | **P3** | **P4** | **P5** |
| --- | --- | --- | --- | --- | --- | --- |
| **FBS** | 0.00 (± 0.20) | 3.20 (± 0.09) | 3.11 (± 0.23) | 4.41 (± 0.42) | 3.57 (± 0.38) | 4.79 (± 0.65) |
| **pHPL** | 0.11 (± 0.16) | 6.20 (± 0.17) | 6.10 (± 0.18) | 6.09 (± 0.04) | 5.98 (± 0.12) | 5.89 (± 0.18) |

## ASC morphology

ASC morphology was assessed using fluorescence microscopy as previously described (Dessels et al., 2018). Briefly, the adherent ASCs were washed with PBS and the cytoplasm and nuclei were stained using 20 µL carboxyfluorescein succinimidyl ester (Cell Trace^®^ CSFE, Thermo Fischer, [Waltham, Massachusetts, USA](https://www.google.co.za/search?espv=2&biw=1920&bih=950&q=Waltham+Massachusetts&stick=H4sIAAAAAAAAAOPgE-LSz9U3MCooMTBJU-IAsTOqjE21tLKTrfTzi9IT8zKrEksy8_NQOFYZqYkphaWJRSWpRcUAAxikqkQAAAA&sa=X&ved=0ahUKEwjuvPb2mMjPAhUrJcAKHU6aBbwQmxMItQEoATAZ)) and 2 µL Vybrant^®^ DyeCycle™ Violet (VDC violet; Invitrogen/Molecular Probes^®^, Life Technologies, Eugene, Oregon, USA), respectively. The ASCs were incubated for 15 minutes at 37˚C in a 5% CO_2_ incubator and subsequently washed with PBS. Micrographs were captured using a 10x magnification objective lens in an AxioVert A1 inverted fluorescence microscope (Carl Zeiss, Gottigen, Germany) equipped with an AxioCam Cm1 camera (Carl Zeiss, Gottigen, Germany). Each image represents an overlay converted from two single channel images, where the first channel (yellow-green fluorescence; filter set 9; excitation: bandpass filter 450-490; emission: longpass filter 515) captured the CSFE staining (visualisation of cytoplasm) and the second channel (DAPI fluorescence; filter set 49; excitation: green 365; emission: bandpass filter 445/50) captured VDC Violet staining (visualisation of nuclei). Images were captured using AxioVision software (Version 4.8.2, Carl Zeiss, Gottigen, Germany) and analysed post-acquisition using Image J imaging software (Version 1.49; Schneider et al., 2012). Images were enhanced for contrast and brightness and nor manipulated.

| 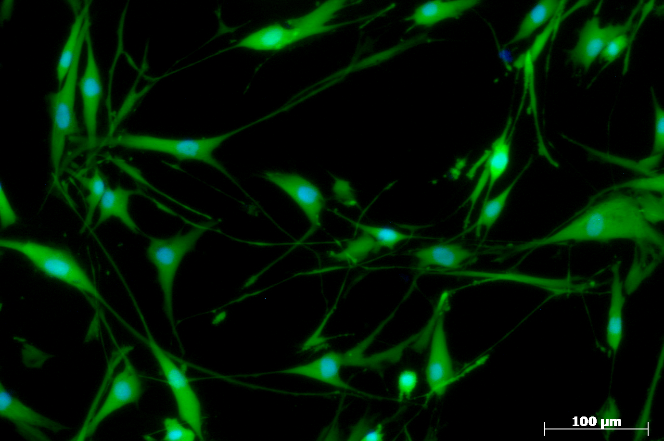  **A** | 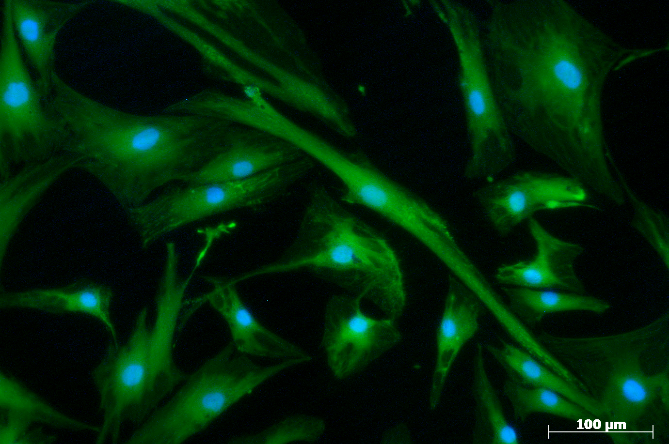  **B** |
| --- | --- |

**Figure 2: Fluorescence micrographs of ASCs expanded in pHPL (A) and FBS (B).** The cytoplasm was stained with carboxyfluorescein succinimidyl ester (Cell Trace^®^ CSFE, Thermo Fischer, [Waltham, Massachusetts, USA](https://www.google.co.za/search?espv=2&biw=1920&bih=950&q=Waltham+Massachusetts&stick=H4sIAAAAAAAAAOPgE-LSz9U3MCooMTBJU-IAsTOqjE21tLKTrfTzi9IT8zKrEksy8_NQOFYZqYkphaWJRSWpRcUAAxikqkQAAAA&sa=X&ved=0ahUKEwjuvPb2mMjPAhUrJcAKHU6aBbwQmxMItQEoATAZ)) and the nuclei were stained with Vybrant^®^ DyeCycle™ Violet (VDC violet; Invitrogen/Molecular Probes^®^, Life Technologies, Eugene, Oregon, USA). Scale bars represent 100 μm.

## Immunophenotype

ASC immunophenotype was determined from the SVF up to and including P5 using a panel of seven fluorochrome-conjugated monoclonal antibodies. The following antibodies were included in the panel: CD73-FITC (eBiosciences, San Diego, USA); CD105-PE (BioLegend, San Diego, USA); CD34 PE-Cy7 (BioLegend, San Diego, USA); CD36 APC (BioLegend, San Diego, USA); CD44 APC/Cy7 (BioLegend, San Diego, USA); CD90 BV421 (BD Biosciences, San Jose, USA) and CD45 BV510 (BD Biosciences, San Jose, USA). At each passage, when confluent, ASCs were dissociated and 100 µL cell suspension was simultaneously stained with 5 µL of each antibody and incubated for 15 minutes in the dark, whereafter 1 mL PBS was added and the tubes centrifuged at 184 *g* for 5 min. PBS was aspirated and 700 µL PBS was added. The cells were analysed on a Gallios flow cytometer (Beckman Coulter, Miami, USA). Unstained cells were used to establish negative limits. The intact ASC population was identified using Forward scatter (FS) vs Side scatter (SS) two parameter plots. All subsequent flow cytometric plots were gated on the intact ASC population. The positive expression of the different surface markers was identified using a one parameter log histogram plot, and the co-expression profiles were obtained using tree plots. Flow cytometry data was analysed post-acquisition using Kaluza Post-Acquisition Flow Cytometry Analysis software (Version 1.3).

Both the ASCs expanded in FBS and pHPL had similar immunophenotypic expression profiles (CD44+CD45−CD73+CD90+CD105+) over the passaging process, where they both reached more than 90% positivity by P3 (Figure 1A). The ASCs expanded in pHPL displayed a more stable (less variable) phenotype at early passages (P0 – P3) compared to ASCs expanded in FBS (Figure 3A & B, P0-P3). Furthermore, both the ASCs expanded in pHPL and FBS were negative for CD31 (endothelial cells; less than 2%) from P3 onwards (Figure 3B).

| **A.**  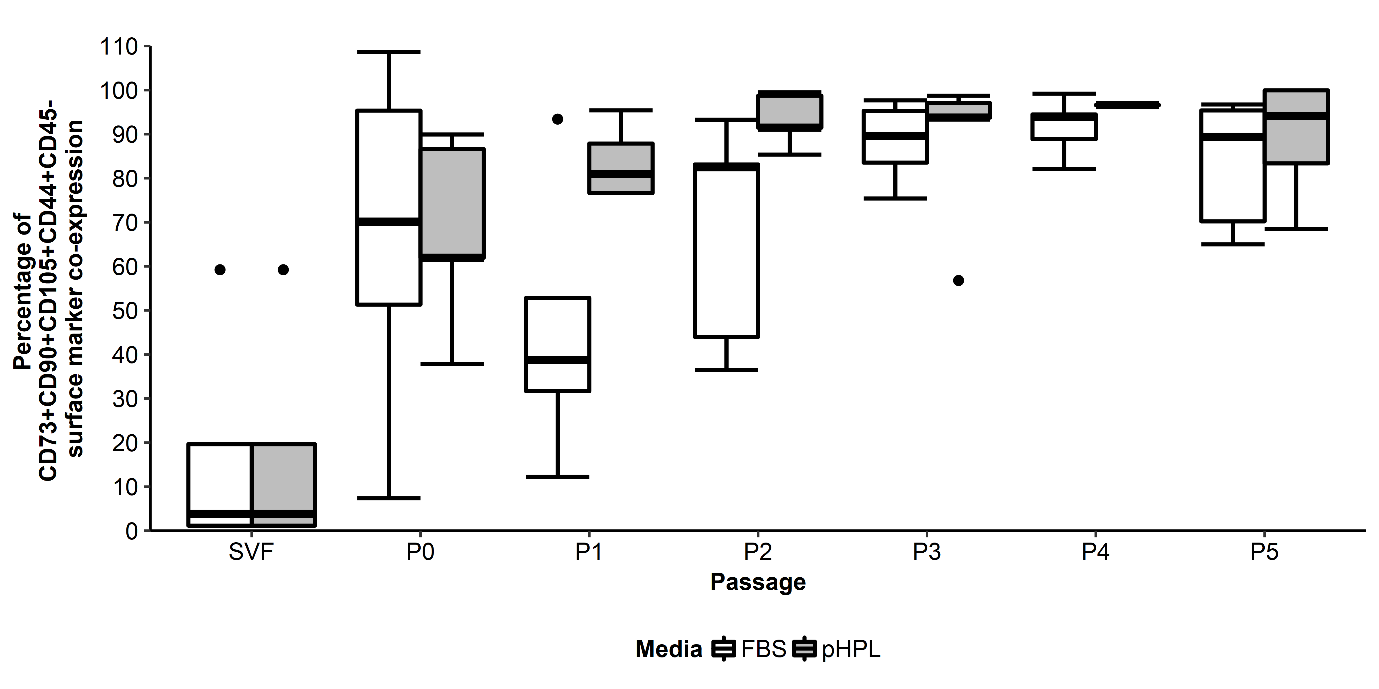 |
| --- |
| **B.**  **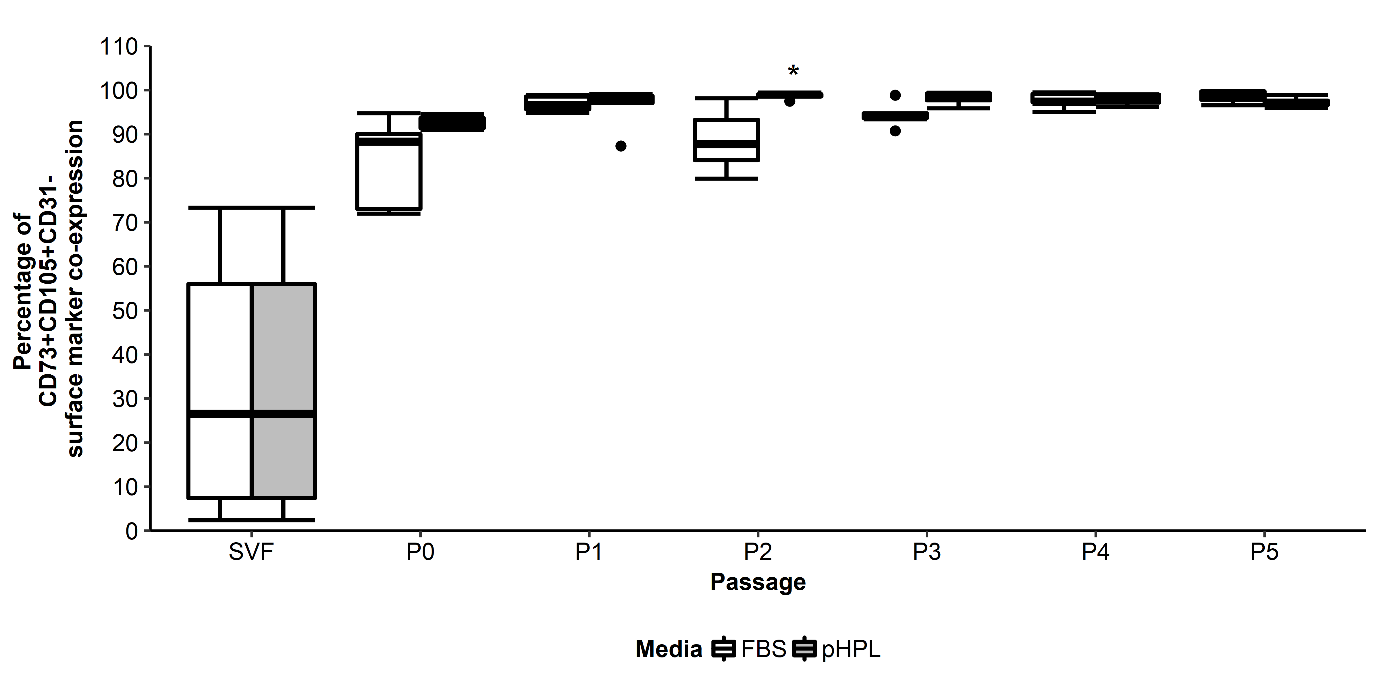** |

**Figure 3: Box and whisker plots representing the co-expression of the CD44+CD45−CD73+CD90+CD105+ (A) and CD31-CD73+CD105+ (B) for ASCs expanded in FBS and pHPL.** Boxes extend from the 1st to 3rd quartiles with the median shown as the solid line intersecting the box, the whiskers extend to the minimum and maximum values that lie within 1.5 x the inter quartile range. Data points beyond the whiskers represent outliers. The sample size is n = 5 and statistical significance is represented by p < 0.05 (*). The white boxes represent the immunophenotype of ASCs expanded in FBS and the grey boxes represent the immunophenotype data of ASCs expanded in pHPL. The significance indicated on the graph compares the immunophenotypic co-expression profile between ASCs expanded in FBS and ASCs expanded in pHPL.

## Adipogenic Differentiation

ASCs cryopreserved at P0 were thawed by adding pre-warmed α-MEM containing either 10% (v/v) pHPL or 10% (v/v) FBS to the cryopreservation tubes. The liquid portion containing the cellular fraction was then transferred to a conical tube (Corning, New York, USA). This step was repeated until the ASCs were completely thawed and all ASCs were transferred. Thereafter the ASCs were centrifuged, seeded into 80 cm^2^ culture flasks and maintained at 37˚C in 5% CO_2_. The passaging process described above was repeated up to P4. At P4, ASCs were dissociated and plated for adipogenic differentiation into both 6-well plates and 80 cm^2^ flasks as described previously [21]. At 80% confluence, ASCs expanded in FBS and pHPL were induced to differentiate into adipocytes by replacing the α-MEM supplemented media with adipogenic induction media consisting of DMEM (DMEM 1x + GlutaMAX™; GIBCO, Thermo Fisher/Life Technologies™, Grand Island, NY, USA), 2% p/s, 1 μM dexamethasone (Sigma-Aldrich Chemie, Steinheim, Germany), 0.5 mM 3-iosbutyl-methylxanthine (Sigma-Aldrich Chemie, Steinheim, Germany), 200 μM indomethacin (Sigma-Aldrich Chemie, Steinheim, Germany) and 10 μg/mL insulin (human recombinant zinc; GIBCO, Thermo Fisher/Life Technologies™, Grand Island, NY, USA) and supplemented with either 10% (v/v) FBS or 5% pHPL. ASCs, both induced and non-induced (controls) were dissociated on the day of induction (day 0), and on days 1, 7, 14 and 21 post induction.

### *Fluorescence microscopy*

Intracellular lipid droplets were visualised by co-staining with Nile Red and VDC Violet (Thermo Fisher Scientific/Life technologies; Waltham, MA, USA) and captured using fluorescence imaging. On the respective days (days 0, 1, 7, 14 and 21) the remaining wells of the 6-well plates were prepared for staining. A working solution of 50 ng/µL Nile Red was prepared in absolute ethanol and 10 µL thereof was added to the wells and carefully mixed by swirling. 2 µL of VDC Violet was then added to each well and again mixed carefully via swirling. The 6-well plates were then incubated at 37°C in 5% CO_2_ for 20 minutes. Thereafter, images were captured with a 20x magnification objective lens using an AxioVert A1 inverted fluorescence microscope equipped with an AxioCam Cm1 camera (Carl Zeiss, Cottigen, Germany). Each image represents an overlay image converted from three single channel images, where the first two channels captured Nile Red staining (visualisation of lipid droplet formation; yellow green fluorescence, filter set 9; and deep red fluorescence, filter set 00) and the last channel captured VDC Violet staining (visualisation of nuclei; DAPI fluorescence, filter set 49). Images were captured using AxioVision software (Version 4.8.2, Carl Zeiss, Cottigen, Germany) and analysed post-acquisition using Image J imaging software (Version 1.49). Images were enhanced for contrast and brightness and were not manipulated.

| 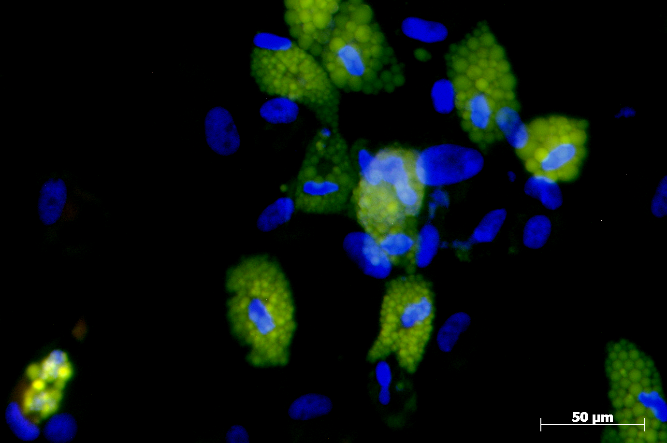  **A** | 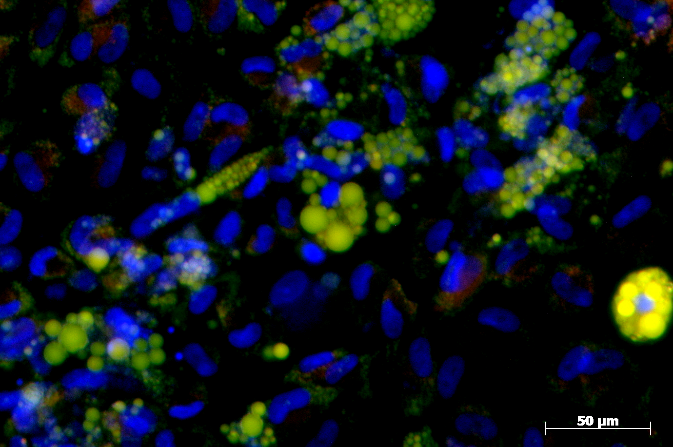  **B** |
| --- | --- |

**Figure 4: Fluorescence microscopy images of day 21 induced A) ASCs expanded in FBS; B) ASCs expanded in pHPL.** Nuclei were stained with VDC Violet and the lipid droplets were stained with Nile Red [50 ng/µL] using protocols previously established in our laboratory [21]. Images were captured with a 20x magnification and the scales represent 50 μm.

| **A.**  **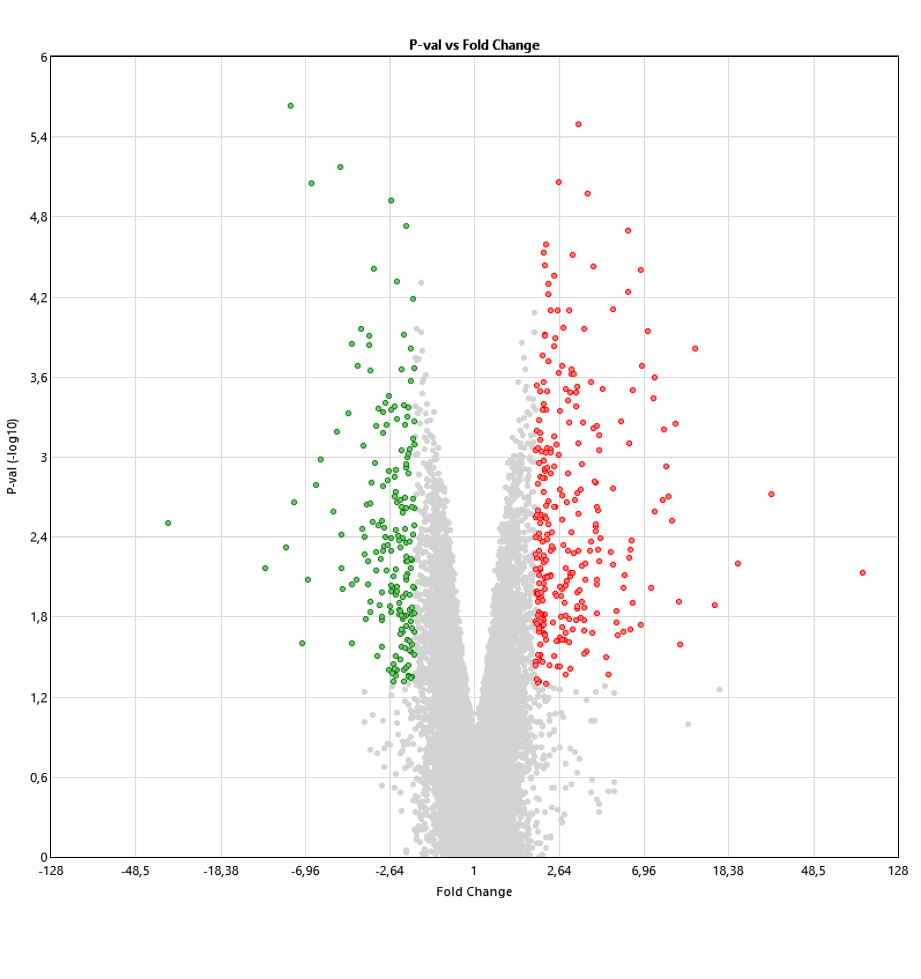** |
| --- |
| **B.**  **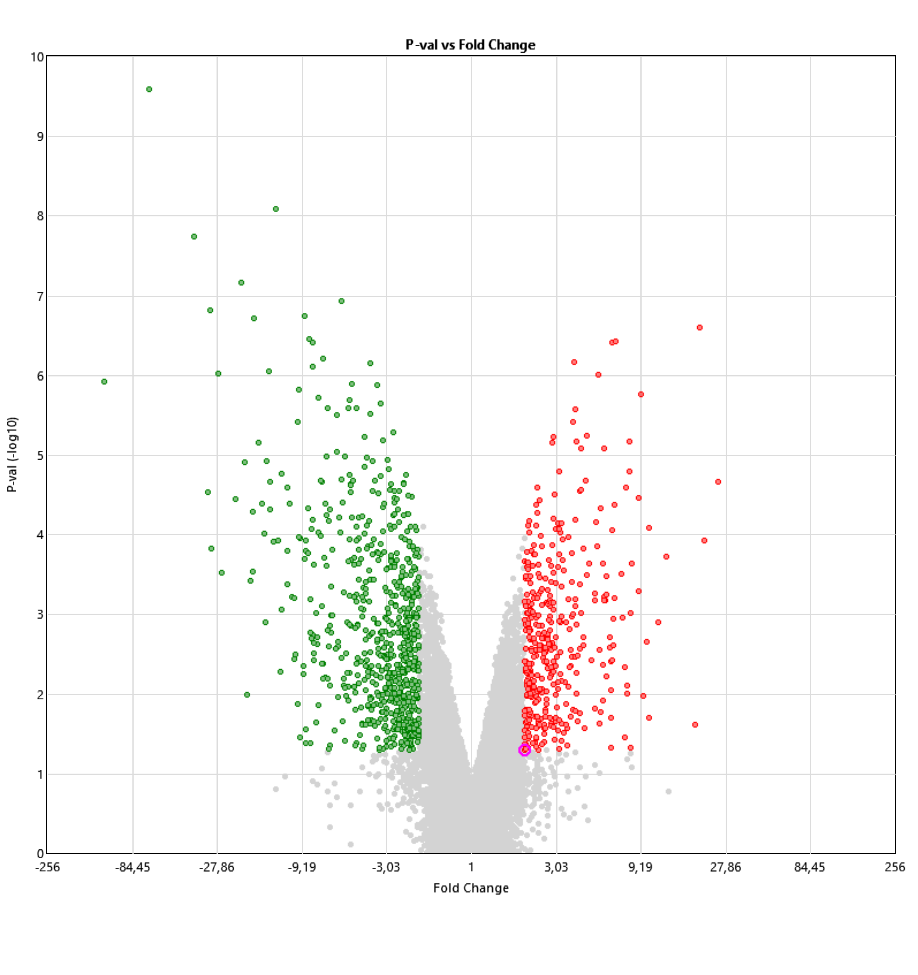** |

| **C.**  **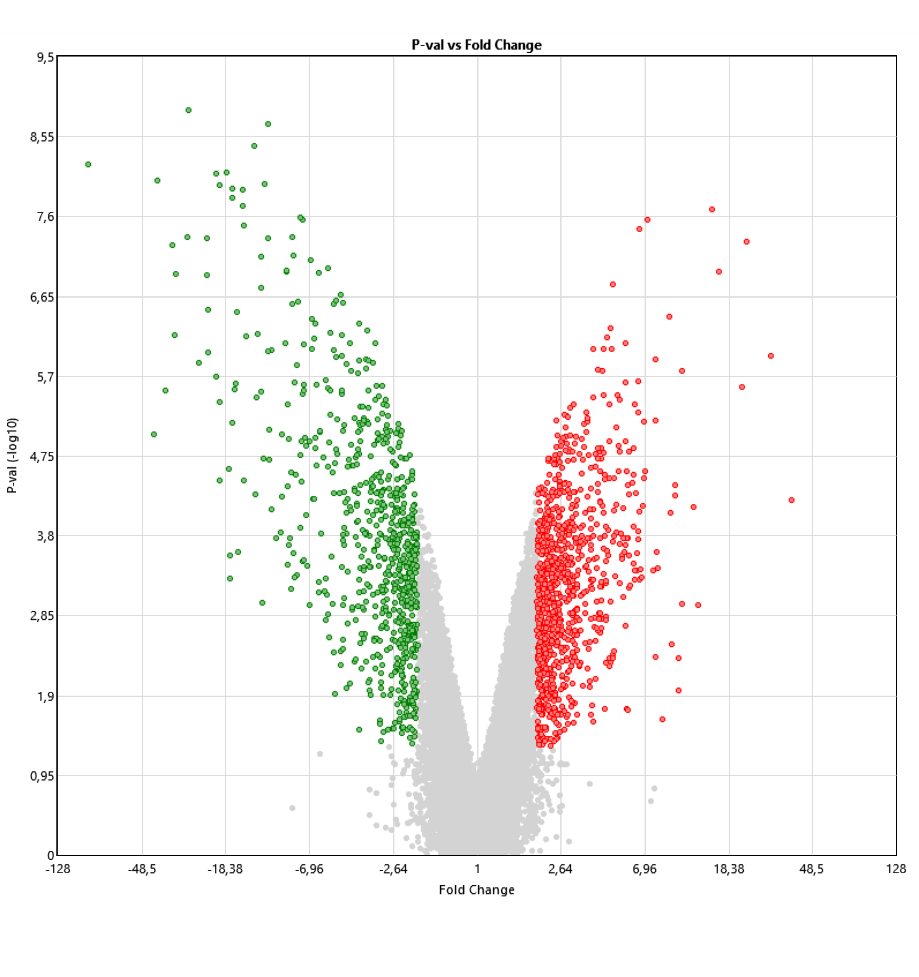** |
| --- |
| **D.**  **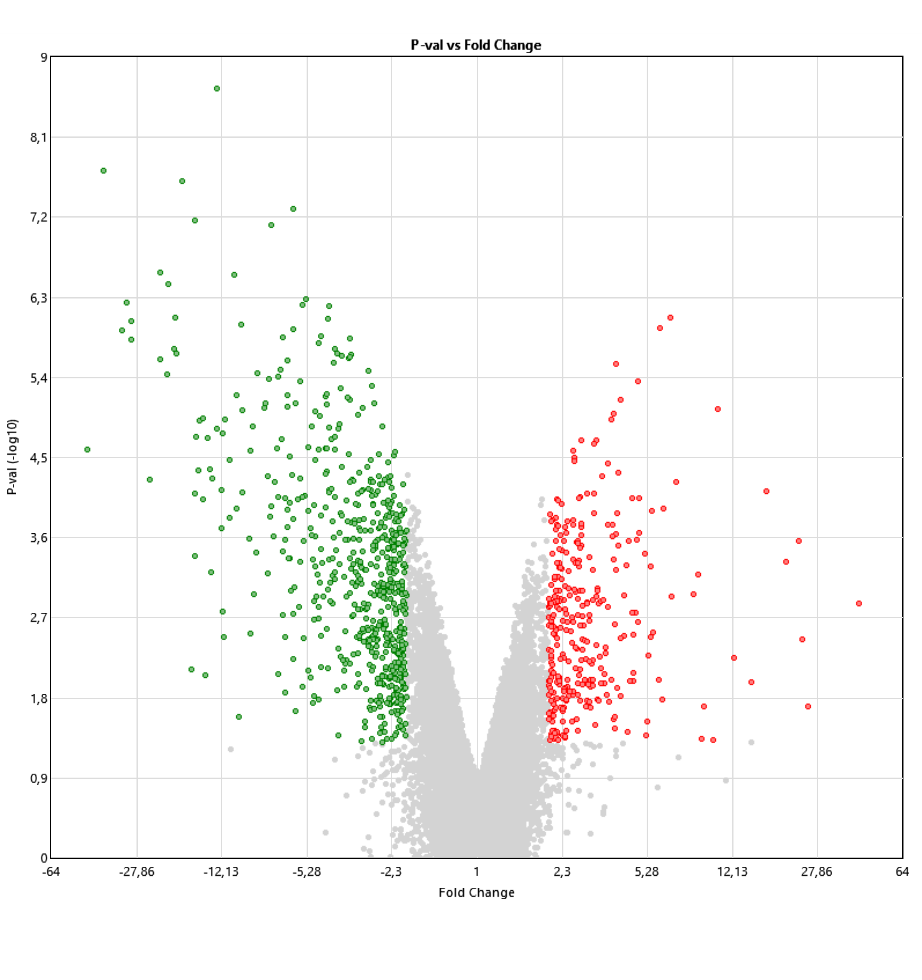** |

| **E.**  **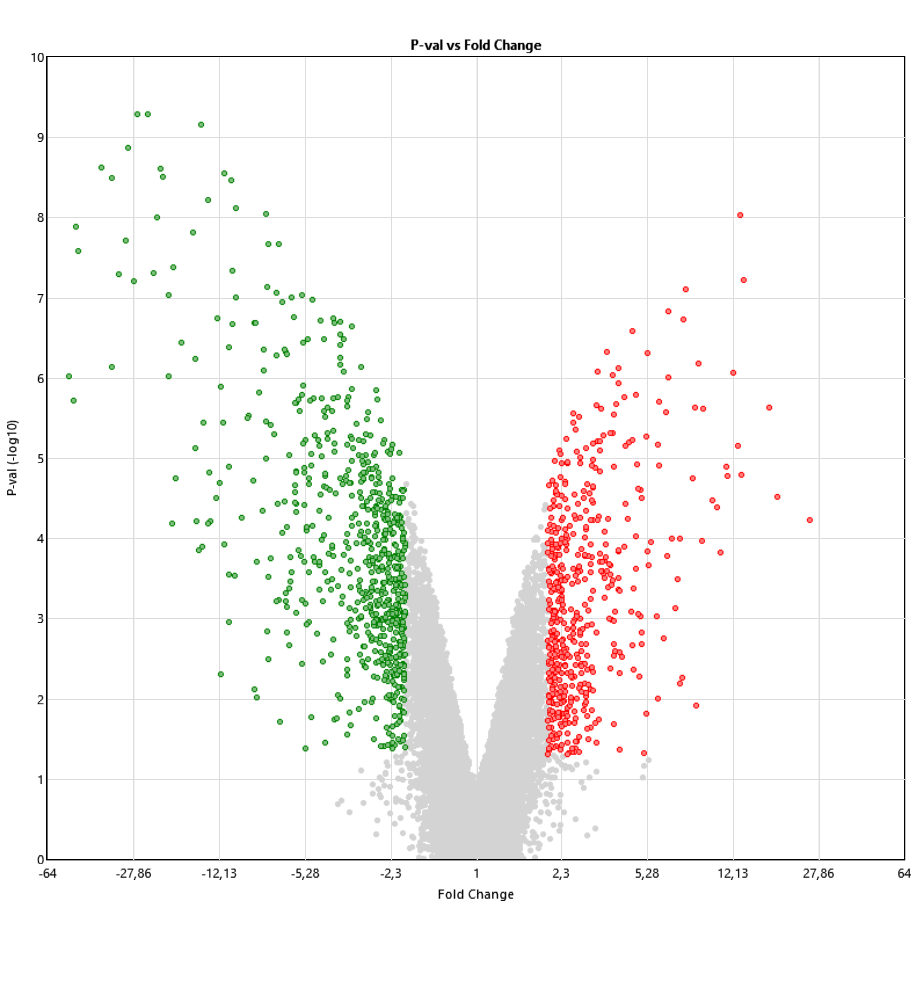** |
| --- |
| **F.**  **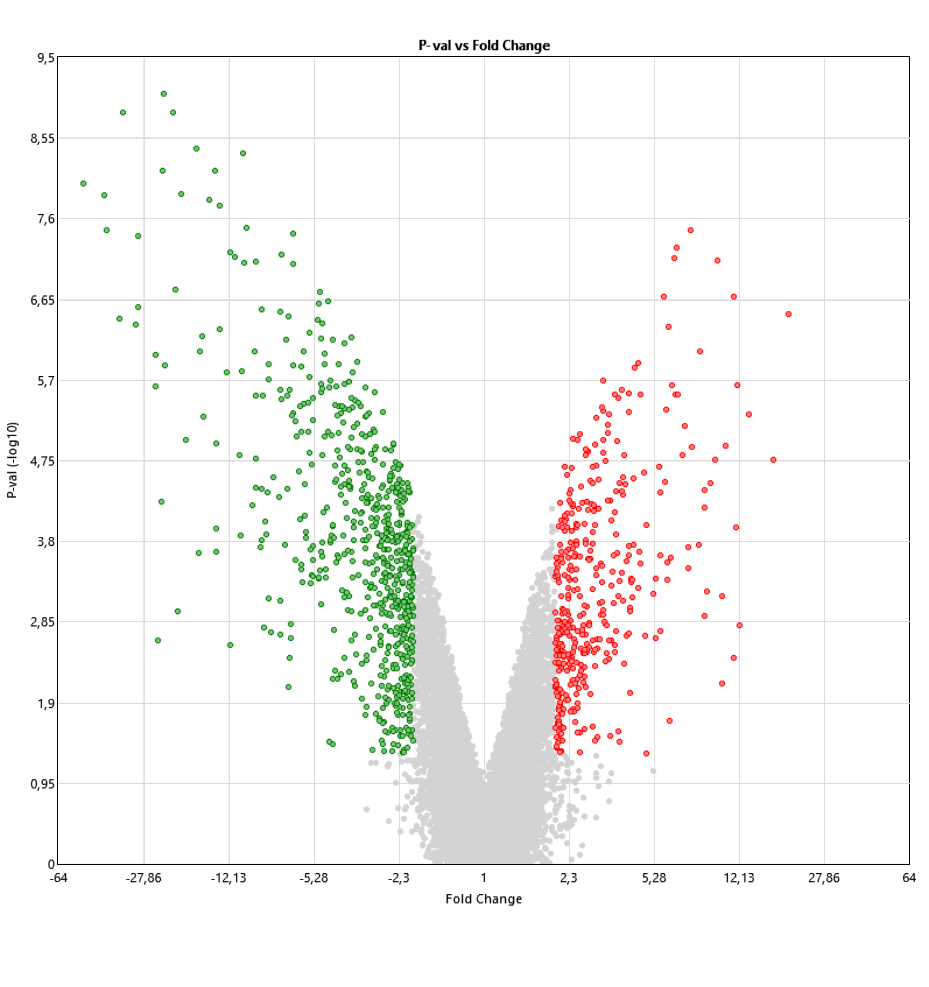** |

**Table 2: Volcano plot of significance (ANOVA p-values) versus fold change showing differentially expressed genes (DEGs) between pHPL and FBS**. A, B, C, D, E and F show DEGs (red dots) between pHPL and FBS at P0, P1, P2, P3, P4 and P5 respectively. The further to the left a gene is, the more downregulated it is while the further to the right a gene is, the more upregulated it is. Meanwhile, the higher a gene is up on the y-axis, the greater it statistical significance. Hence, a gene at the top-left and top-right hand corner of each plot will represents the most significantly downregulated and upregulated gene respectively between pHPL and FBS at each passage.
